# Association between neurodevelopmental disorders in congenital heart disease and changes in circulatory metabolites and gut microbiota composition

**DOI:** 10.3389/fmicb.2025.1639057

**Published:** 2025-07-30

**Authors:** Jia An, Qiang Wang, Zihao Bai, Siyu Ma, Zhaocong Yang, Di Yu, Xuming Mo

**Affiliations:** ^1^Nanjing Children’s Hospital, Clinical Teaching Hospital of Medical School, Nanjing University, Nanjing, China; ^2^Department of Cardio-Thoracic Surgery, Children’s Hospital of Nanjing Medical University, Nanjing, China

**Keywords:** neurodevelopmental disorders, gut microbiota, metabolites, congenital heart disease, infant

## Abstract

**Background:**

Neurodevelopmental disorder (ND) has emerged as a critical factor affecting the long-term quality of life among patients with congenital heart disease (CHD). The aim of this study was to provide a multi-omics perspective on the mechanisms of ND.

**Methods:**

We analyzed the serum metabolome and gut microbiome of children with ND and non-ND (NND) in CHD populations.

**Results:**

In this prospective observational study, we identified associations between serum metabolites, gut microbial, and ND. Linolenic acid was most closely related to neurodevelopmental outcomes, showing positive correlations with multiple neurodevelopmental domains. Among the gut microbiota, the Escherichia genus was most strongly associated with neurodevelopmental outcomes, and negative correlations with neurodevelopmental domains.

**Conclusion:**

This multi-omics study reveals significant association between altered serum metabolites, gut microbiota dysbiosis, and neurodevelopmental outcomes in children with CHD. The microbes and metabolites identified here may contribute to addressing the challenge of ND in the CHD population. Based on our findings, therapeutic strategies to reduce the risk of ND could be developed, including targeted manipulation of the gut microbiota and metabolites.

## Introduction

Congenital heart disease (CHD) is the most common birth defect, affecting 1–1.2% of babies born each year (Pierpont et al., 2018). Following advances in surgical and cardiological therapy, more than 90% CHD now survived to adulthood (Mandalenakis et al., 2020), resulting in greater appreciation and interest in long-term health outcomes, such as neurodevelopment. Neurodevelopmental disorder (ND) is now recognized as a lifespan issue for many CHD children especially in cyanotic CHD (CCHD) (Marino et al., 2012). More than 50% CHD demonstrate mild to moderate ND and later cognitive impairment (Verrall et al., 2021). Early manifestations of ND typically included poor feeding, speech and language delay, challenges with gross and fine motor movement, and early cognitive concerns, and the decline in communication, social–emotional and executive functioning in older children (Huisenga et al., 2021). Identifying ND risk in CHD is challenging. Macro and microstructural changes in the brain can help in the early identification of ND, the fact is that proposition of critically ill were difficulty to finished the brain MRI, and these patients are exactly the ones who are at high risk for ND. Therefore, developing new biomarkers for ND is necessary. With the development of microbiome and metabolomics bring the opportunity to characterize the levels of a various microbiota and metabolites.

A growing body of evidence shows that the early life gut microbiome is a crucial modular involved in immune system training and modulation as well as in host health (Fan and Pedersen, 2021). The microbiome appears to be an important biological player mediating the impact of environmental factors on child neurodevelopmental and health. A large body of evidence emphasis the gut nerves system and the relationship between the gut microbiota and the brain, which known as the “gut-brain axis” (Gusella et al., 2021). Previous studies have reported altered gut microbiota in patients with central nervous system (CNS) diseases [e.g., autism spectrum disorder (ASD) (Strati et al., 2017), Alzheimer’s disease (Ferreiro et al., 2023), Huntington’s disease (Qian et al., 2024), etc.]; animal experiments have demonstrated that altered gut microbiota may be involved in the genesis and progression of CNS (Mossad et al., 2022; Olson et al., 2021). Currently, several researches demonstrate differing gut microbiome and metabolic patterns in ND as compared to health controls (Ahrens et al., 2024; Tamana et al., 2021). The impact of dysbiosis on the host can be dramatic but unnoticeable during critical periods of gut microbiota development.

And yet, compositional and functional alternations in gut microbiota and metabolomic and their correlation with neurodevelopmental outcomes in patient with CHD remain unclear. In this study, we performed 16S rRNA sequencing and metabolome analyze in CHD cohorts, aim to explore the correlation between alternated gut microbiota, metabolome and ND.

## Materials and methods

### Study population and sample collection

This prospective observational study was approved by the Ethics Committee of Nanjing Children’s Hospital (No. 20191225412) and informed consent was obtained from all study participants. From January 2019 to January 2022, patients admitted to the Department of Cardio-thoracic Surgery at Nanjing Children’s Hospital and diagnosed with CHD according to the ICD-10 codes, and age at surgery within the first year of life were enrolled in this study. Patients with a genetic disorder, visual and hearing problems, metabolic disorder, gastrointestinal disease or took antibiotics, probiotics within 2 weeks were excluded. Informed consent was obtained from all the guardians of the participants for sample and data collection. The basic information collected as follows: age at surgery, SpO_2_, gender, weight, born weight, born model (vaginal or cesarean), Apgar score, breastfeeding status (partially or not breastfeed), and maternal education; heart function index left ventricular ejection fraction (LVEF) and left ventricular Fraction shortening (LVFS); surgical procedure information including the risk adjusted classification for congenital heart surgery-1 (RACHS-1) score, duration of cardiopulmonary bypass (CPB) and aortic cross clamp (ACC); prognostic information, including length of stay (LOS) in the intensive care unit (ICU) and hospital. Fecal and blood were collected on admission. Plasma was separated and immediately stored at −80°C.

### Neurodevelopment assessment

The Ages and Stages Questionnaire, Third Edition (ASQ-3) is a parent-completed questionnaire to assess the overall development of children aged 1 to 66 months. Each questionnaire includes 30 items covering five developmental skill domains: communication, gross motor, fine motor, problem solving, and personal-social. Responses are summed to give a score of 0 to 60 for each domain and an overall maximum 300. Analyses were based on domain specific scores, using established screening cut-off points: an ASQ score below threshold was defined as a score lower than 2 standard deviations from the mean on any of five domains (Pierrat et al., 2017), which would identify ND.

### Shotgun metagenomics sequencing

Microbial community genomic DNA was extracted from fecal samples using the E.Z.N.A.^®^ soil DNA kit (Omega Bio-tek, Norcross, CA, United States). The quality and concentration of DNA were determined by 2.0% agarose gel electrophoresis and purified using the AxyPrep DNA Gel Extraction Kit (Axygen Biosciences, Union City, CA, United States). Purified DNA was used for PCR amplification of the 16S rRNA gene of distinct regions V3-V4. Purified amplicons were pooled in equimolar and paired-end sequenced on an Illumina MiSeq PE 300 platform/NovaSeq PE250 platform (Illumina, San Diego, United States).

Raw FASTQ version 0.19.6 sequence files were collected using a perl script and then quality-filtered by Fastp version 0.19.6 and merged by Flash version 1.2.11 with the following strict criteria: quality threshold 20, minimum overlap 10 bp and maximum mismatch ratio 0.2. Then the optimized sequences were clustered into operational taxonomic units (OTUs) using UPARSE version 7.0 with 97% sequence similarity level. The most abundance sequence for each OTU was selected as a representative sequence. OTU consisting of only a single sequence were removed. The taxonomy of each OTU representative sequence was analyzed by RDP Classifier version 2.2 against the 16S rRNA gene database using confidence threshold of 0.7.

### Non-targeted metabolomics analysis

Non-targeted metabolomics analysis was performed as previous described (Olson et al., 2021). In summary, metabolomics is conducted using the LC-MS analysis platform. Samples undergo preprocessing to remove proteins and impurities and extract metabolites. Then, metabolites are monitored in both positive and negative modes in LC-MS, generating MS and MS/MS information. Progenesis QI software (Waters Corporation, Milford, United States) is used for metabolite annotation and data preprocessing, ultimately resulting in a list of metabolites and a data matrix.

### Statistical analysis

All statistical analysis were performed in R software (version 4.2.2). Shapiro–Wilk test was performed to evaluated the normal distributed for continuous data, data will show as mean ± standard deviation (SD) if normally distributed, or as median and interquartile range if non-normally distributed. Differences between two groups were assesses using Student’s *t*-test or Wilcoxon rank-sum test. Binary variables were shown as frequency and percentage and compared using chi-square test. *p*-value <0.05 was considered statistically significant.

### Gut microbiota

Comparison of the alpha diversity (Chao and Shannon indexes) at the OTU level by using Wilcoxon test. Principal coordinates analysis (PCoA) of the Bray–Curtis distance metric was performed to reflect the structure of microbial in each cohort, and dissimilarities were revealed through analysis of similarities (ANOSIM) method. Linear discriminant analysis effect size (LEfSe) with a linear discriminant analysis (LDA) log10 threshold 3.0 for identify of discriminative taxonomic feature. The permutational multivariate analysis of variance (PERMANOVA) is a method of decomposing the total variance according to the Bray–Curtis distance, which can be used to analyze the explanatory power of different clinical factors for gut bacterial variation.

### Metabolites

The significant differences among cohorts were calculated based on Student’s *t*-test. Benjamini–Hochberg false discovery rate (FDR) was calculated as well as fold changed (FC). The biochemical pathways of differential metabolites were identified by searching the KEGG database and classified by their pathway involvement.

### Model construction

In this study, we employed the Random Forest algorithm to identify key microbial taxa and serum metabolites. First, the data were used as the feature matrix, with sample labels as the response variable. Through bootstrap sampling and random feature selection, 500 decision trees were constructed. At each node split, a subset of features was randomly selected to mitigate overfitting. The final predictions were obtained by aggregating the results of all trees using majority voting for classification. Feature importance was assessed by calculating the mean decrease in impurity for each feature across all trees, and model performance was evaluated using cross-validation. Based on the ranking of feature importance, the microbial taxa contributing most to the classification task were identified.

Spearman’s correlation analysis was employed to clarify associations between the abundance of metabolites, bacterial abundance, and ASQ scores.

Finally, univariate and multivariate logistic regression models was used to assess the association between bacteria, metabolite, and neurodevelopment outcome. Regression coefficients, odds ratios (ORs), and 95% confidence intervals (CIs) were calculated, with statistical significance evaluated using the Wald test. Additionally, the predictive performance of the factor was assessed by plotting the receiver operating characteristic (ROC) curve and calculating the area under the curve (AUC).

## Results

From January 2019 to January 2022, 68 infants with CHD were enrolled in the neurodevelopmental-microbiome-metabolomic cohort. The mean (SD) age was 6.44 (2.65) months, with 38 (55.88%) males and 30 (44.12%) females. Neurodevelopmental assessments were conducted for all enrolled infants using the ASQ-3 score. Infants scoring below 2 SD in any domain of the ASQ were classified into the ND group, while the others were classified into the non-ND (NND) group. Twenty-five infants (37%) were identified as ND. Age at surgery, gender, BMI *z*-score, prematurity, Apgar score at 5 min, born weight, and breastfeeding showed no significant differences between the ND and NND groups. However, the ND group had lower SpO_2_ levels and lower maternal education levels compared to the NND group (*p* < 0.05), as shown in Table 1. Multivariate logistic regression, adjusted for age at surgery, gender, BMI *z*-score, and prematurity, revealed that ND was associated with SpO_2_ (OR = 0.89, 95% CI: 0.80–0.97, *p* = 0.04) but not with maternal education level (OR = 0.38, 95% CI: 0.11–1.09, *p* = 0.08; Supplementary Table S1). Linear regression analysis revealed that, after adjusting for confounding factors, SpO_2_ was positively associated with the ASQ total score, communication, and gross motor skills, whereas maternal education level was positively linked to fine motor, problem-solving, and personal-social skills (Supplementary Table S2).

**Table 1:** Demographic data and clinical characteristic of subgroup based on neurodevelopmental outcome.

Characteristics	NND (*n* = 43)	ND (*n* = 25)	*p*-value
CCHD, *n* (%)	15 (34.88%)	14 (56%)	0.15
Female, *n* (%)	16 (37.21%)	14 (56%)	0.21
Gestational age <37 weeks, *n* (%)	8 (18.6%)	3 (12%)	0.73
Apgar score at 5 min	8 (8, 9)	9 (7, 9)	0.88
Born weight, kg	3 (2.65, 3.40)	3 (2.40, 3.37)	0.55
Age at surgery, months	6.24 (2.58)	6.78 (2.79)	0.42
BMI *z*-score	−0.67 (2.54)	−0.06 (1.32)	0.19
Born mode, *n* (%)			
Vaginal	19 (44.19%)	19 (76%)	0.02
Cesarean	24 (55.81%)	6 (24%)	
Breastfeeding, *n* (%)			
None	26 (60.47%)	10 (40%)	0.17
Partial	17 (39.53%)	15 (60%)	
Maternal education			
Less than high school	3 (6.98%)	6 (24%)	0.02
High school or some college	26 (60.47%)	17 (68%)	
College graduate or more	14 (32.56%)	2 (8%)	
*ASQ at admission*			
ASQ total score	246.28 (30.82)	175.20 (27.82)	<0.001
Communication	48.26 (8.30)	40.80 (12.39)	0.01
Gross motor	45.93 (10.76)	28 (11.18)	<0.001
Fine motor	55 (50, 60)	40 (35, 50)	<0.001
Problem solving	55 (47.5, 60)	35 (30, 45)	<0.001
Personal-social	50 (40, 60)	25 (20, 40)	<0.001
SpO_2_, %	96.91 (3.56)	91.88 (9.39)	0.002
LVEF, %	67.98 (67, 69.9)	67.3 (66.2, 68.5)	0.52
LVFS, %	36.80 (35.60, 38.30)	36.40 (35.60, 37.2)	0.46

Follow-up period			
Characteristics	NND (*n* = 35)	ND (*n* = 18)	*p*-value
Age at follow-up, months	21.29 (3.84)	21.27 (3.63)	0.99
Maternal education			
Less than high school	4 (11.43%)	2 (11.11%)	0.91
High school or some college	21 (60%)	12 (66.67%)	
College graduate or more	10 (28.57%)	4 (22.22%)	
RACHS-1			
1	0	0	0.55
2	32 (91.43%)	15 (83.33%)	
3	2 (5.71%)	3 (16.67%)	
4	1 (2.86%)	0	
5	0	0	
6	0	0	
Aortic cross clamp time, minutes	37 (29, 47)	43 (35, 61)	0.31
Cardiopulmonary bypass time, minutes	63 (46.5, 79)	69.5 (54, 96)	0.45
LOS of ICU, days	6 (5, 8)	6.5 (5, 8)	0.87
LOS of hospital, days	23 (21, 26.5)	24 (22, 28)	0.48
ASQ total scores	227.71 (42.05)	209.44 (250.9)	0.17
Communication	46.57 (8.47)	43.89 (13.35)	0.45
Gross motor	42 (11.52)	36.11 (16.14)	0.13
Fine motor	50 (40, 55)	45 (40, 55)	0.58
Problem solving	50 (37.5, 60)	45 (35, 55)	0.32
Personal-social	40 (35, 55)	37 (25, 55)	0.29

NND, non neurodevelopmental disorders; ND, neurodevelopmental disorder; CCHD, cyanotic congenital heart disease; BMI, body mass index; ASQ, Ages and Stages Questionnaire; LVEF, left ventricular ejection fraction; LVFS, left ventricular fraction shortening; RACHS-1, the risk adjusted classification for congenital heart surgery-1; LOS, length of stay; ICU, intensive care unit.

### Characteristic gut microbiota in ND

**Figure 1:**
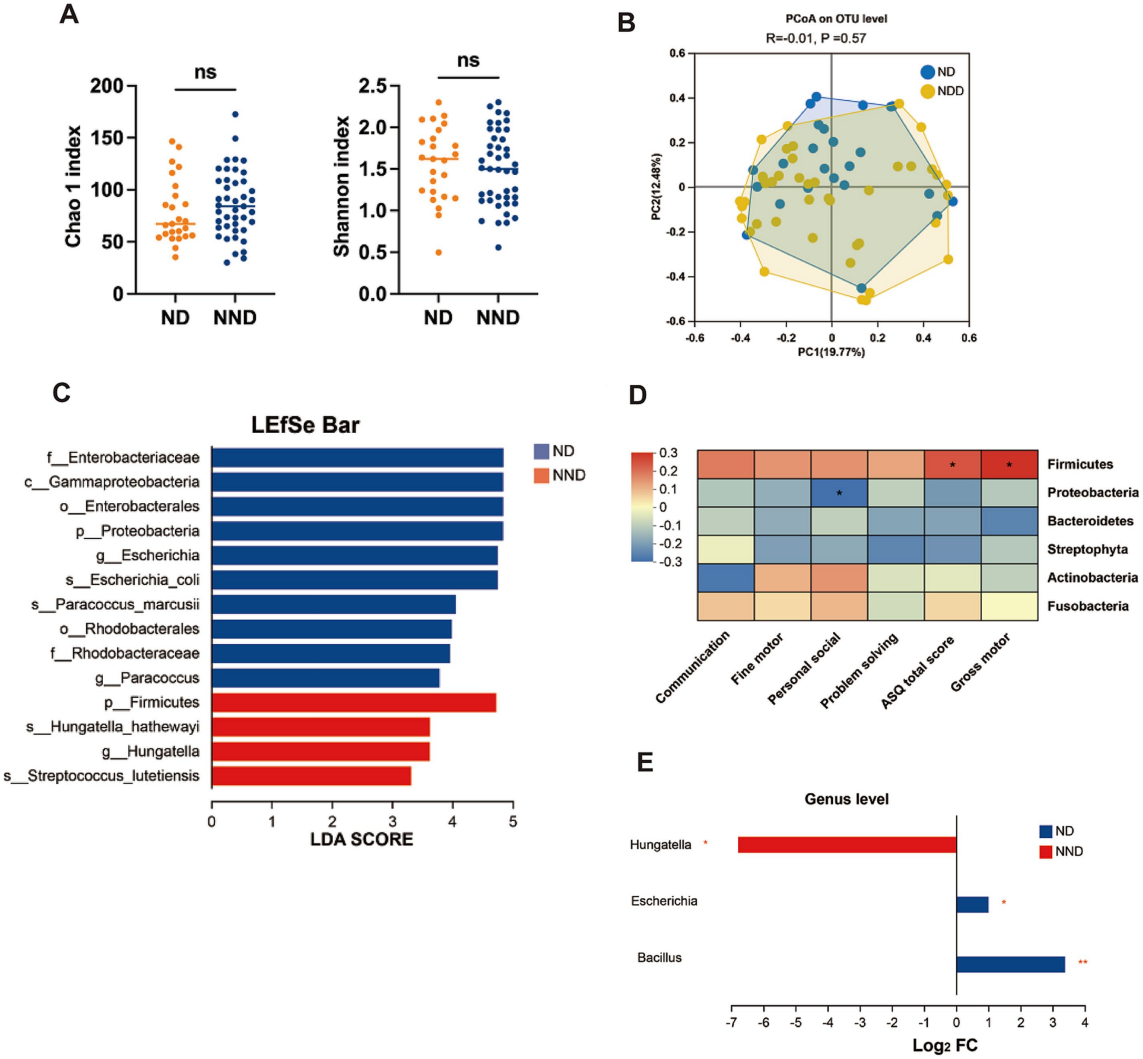
Comparison of gut microbiome alterations in neurodevelopment disorder (ND) and non-ND (NND) groups. **(A)** Boxplots of alpha diversity indices (Wilcoxon test). **(B)** PCoA score plot based on 16S rRNA sequencing microbial taxonomy data. Each points represent a sample and the colors represent different groups. The difference in microbiome composition were assessed by ANOSIM and Bray–Curtis distance. **(C)** LEfSe used to identify essential differences in bacterial abundance (phylum to species level) between the ND and NND groups. Only taxa with a significant LDA threshold value >3 are shown. **(D)** Spearman’s correlation heatmap representing relationships between ASQ-3 scores and the abundance of taxa at phylum level. Distinct colors represent correlation levels; “*” indicates a reliable correlation. **(E)** At the genus level, the bacteria showing significant differences between the ND and NND groups were screened based on the criteria of fold change >2 and *p*-value <0.05.

To investigate whether there are differences in gut microbial structures among patients with varying neurodevelopmental outcomes, we collected fecal samples and characterized the gut microbiota composition using high-throughput 16S rRNA amplicon sequencing. After quality filtering, 3,303,576 reads were obtained, revealing a gut microbial profile consisting of 674 OTUs. At the OTU level, no significant differences in alpha and beta diversity were observed between the ND and NND groups (Figures 1A,B). To further explore the differences in microbiota composition between the two groups, we performed differential analysis using LEfSe at multiple taxonomic levels, with LDA scores used to measure the effect size of species contributing to the differences. We found that the ND group was predominantly characterized by the Proteobacteria phylum (LDA >4, *p* = 0.033), while the NND group was enriched with the Firmicutes phylum (LDA >4, *p* = 0.031), as shown in Figure 1C. Additionally, we confirmed that the Firmicutes phylum was positively correlated with the ASQ total score and gross motor skills, whereas the Proteobacteria phylum was negatively correlated with personal-social skills (Figure 1D). At the genus level, three differentially abundant genera were identified with a fold change >2 and an adjusted *p*-value <0.05. The Hungatella genus was enriched in the NND group, while the Bacillus and Escherichia genera were enriched in the ND group (Figure 1E). The Hungatella genus is associated with short-chain fatty acid (SCFA) production, participates in bile acid metabolism regulation, and modulates the immune system through its metabolites. Certain strains of Bacillus and Escherichia can produce gamma-aminobutyric acid (GABA), thereby influencing neurodevelopmental processes.

**Figure 2:**
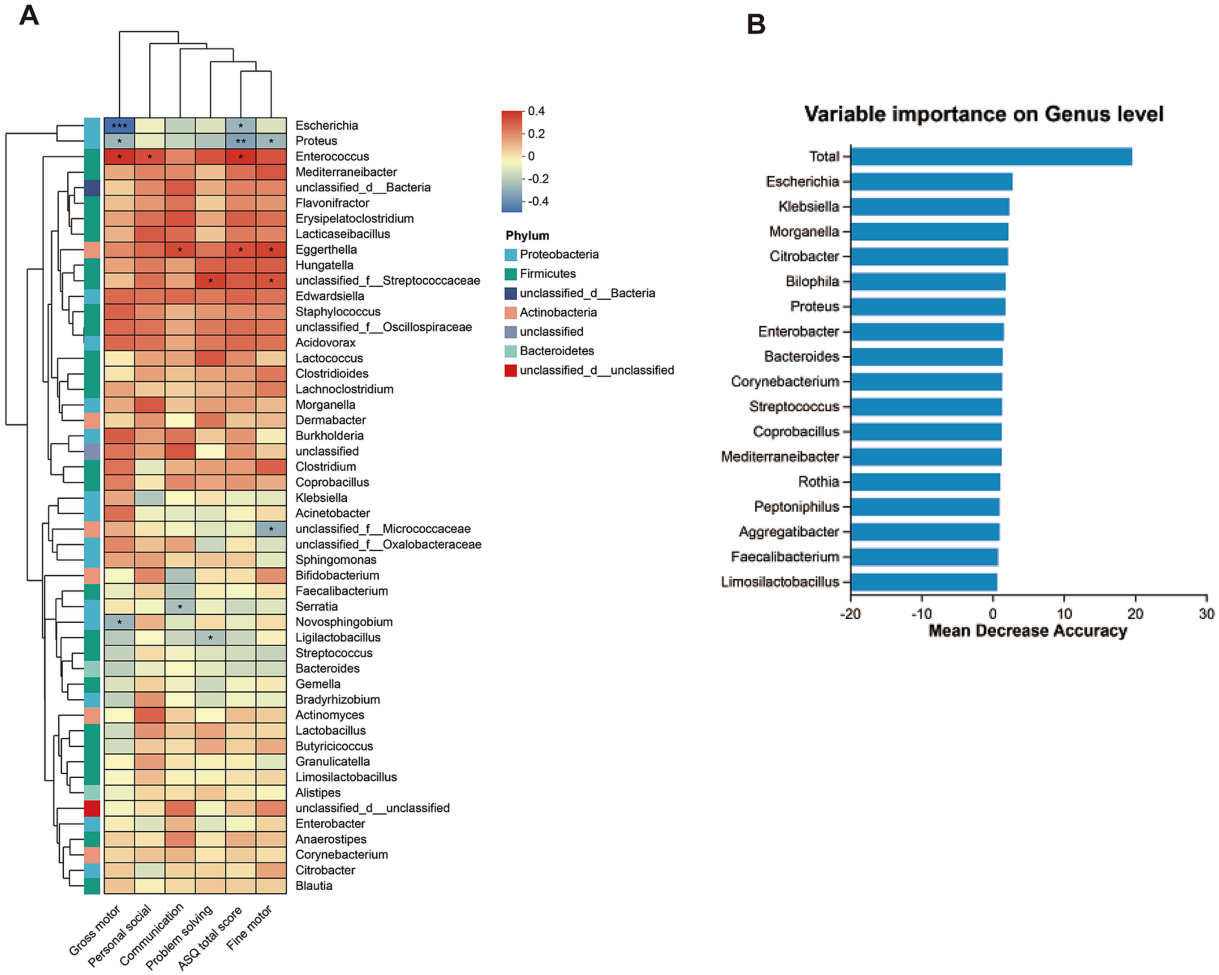
The correlation of genus bacteria and neurodevelopmental outcomes. **(A)** Heatmap representation of spearman correlation between ASQ-3 scores and dominant bacteria at genus level (top 50). **(B)** The random forest algorithm calculates the dominant bacterial genera related to neural development outcomes.

Correlation analysis was conducted to uncover the associations between differentially abundant genera and ASQ scores. At the genus level, spearman analysis revealed that the Escherichia genus was negatively correlated with ASQ total and gross motor scores, the Proteus genus was negatively correlated with ASQ total, gross motor, and fine motor scores, and the Enterococcus genus was positively correlated with gross motor, personal-social, and ASQ total scores. Whereas the Hungatella genus showed no correlation with ASQ scores (Figure 2A). To identify the bacteria most influential on neurodevelopment, we constructed a disease diagnosis model based on random forest analysis, which identified the Escherichia genus as the most significant bacterium (Figure 2B and Supplementary Table S3).

Additionally, PERMANOVA analysis determined that the overall microbial composition differences among individuals were associated with age at surgery and SpO_2_ level but not with gender, BMI *z*-score, born model, prematurity, or breastfeeding (Supplementary Table S4).

### Characteristic serum metabolic profiles in ND

**Figure 3:**
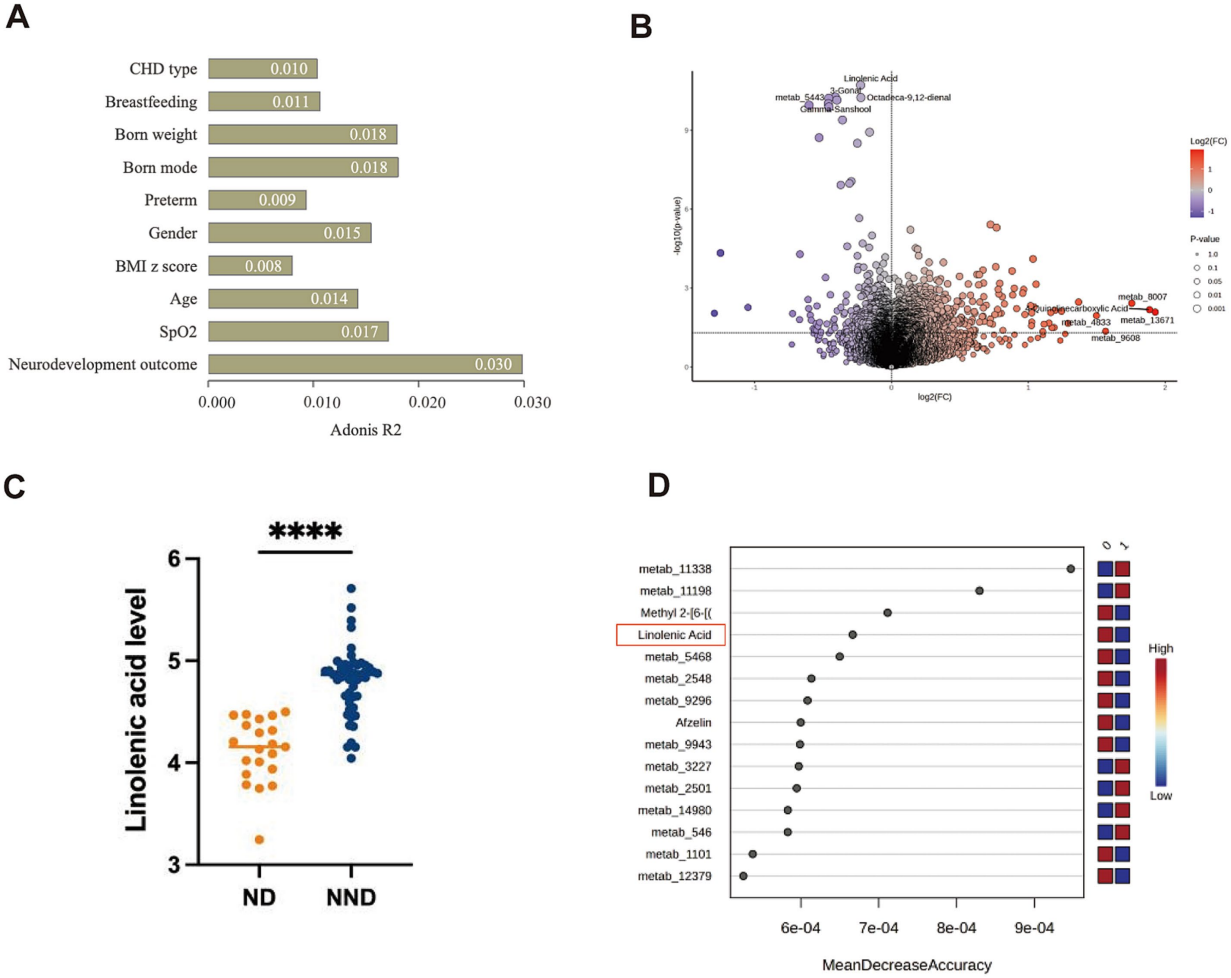
Comparison of serum metabolites alterations in neurodevelopment disorder (ND) and non-ND (NND) groups. **(A)** PERMANOVA analysis of covariates influencing the serum metabolomic profiles of the patients ranked by impact (*R*^2^ value). *p* < 0.05 is considered to have a significant impact. **(B)** A volcano plot showing significant changes in metabolites between different neurodevelopment outcomes. Blue indicates that the metabolite is higher in the NND group, red indicates that the metabolite is higher in the ND group, and the top five metabolites are labeled. According to the Wilcoxon test, a *p* < 0.05 indicates that the difference is statistically significant. **(C)** Comparison of the specific quantities of linoleic acid between the ND group and the NND group. **(D)** The random forest algorithm calculates the dominant serum metabolites related to neural development outcomes. The “0” represent the NDD group, and the “1” represent the ND group.

Serum samples were not collected from four patients in the ND group, leaving a total of 64 patients who completed untargeted serum metabolomic sequencing. PERMANOVA was used to assess the contributions of age at surgery, gender, BMI *z*-score, breastfeeding, and neurodevelopmental outcomes to metabolomic variability. Although neurodevelopmental outcomes contributed the most to the variability, no clinical characteristics were found to have a significant impact on the differences (all *p*-values >0.05, Figure 3A). Unsupervised PCA analysis showed that the metabolomic profiles of ND and NND groups could not be significantly distinguished in the first and second dimensions. Nevertheless, 1,386 metabolites were found to differ between the ND and NND groups (*p* < 0.05, Figure 3B and Supplementary Table S5), with 31 metabolites remaining significant after correcting for the FDR (Supplementary Table S6). We observed that linolenic acid (Figure 3C), gamma-sanshool, and octadeca-9,12-dienal were significantly decreased in the ND group, while 4-quinolinecarboxylic acid was higher in the NND group. Using a decision tree-based random forest algorithm, metabolites were ranked based on their sensitivity in distinguishing the groups, with the highest-scoring metabolites identified as key metabolites. Random forest analysis confirmed linolenic acid as a critical metabolite associated with neurodevelopment outcomes (Figure 3D).

**Figure 4:**
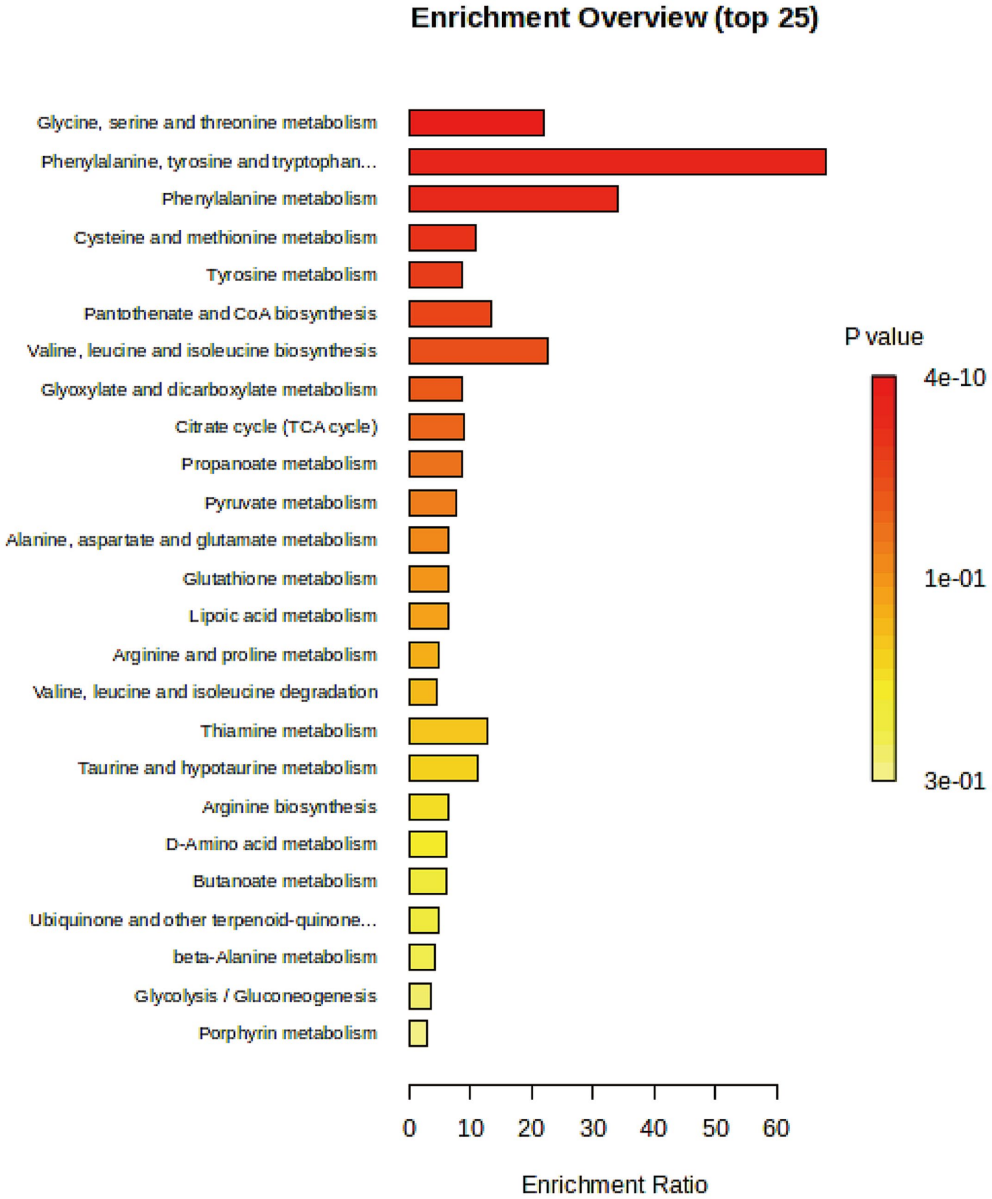
Pathway enrichment analysis of significantly changed metabolites with enrichment ratio on the *x*-axis and bars color-coded by *p*-value.

We conducted a pathway enrichment analysis on the significantly altered metabolites to summarize the broad metabolic changes during neurodevelopment. As shown in Figure 4, 14 pathways exhibited significant changes. The differential metabolites appeared to be heavily involved in amino acid metabolism, such as glycine, serine, threonine, phenylalanine, and tyrosine metabolism, as well as energy metabolism, including pyruvate metabolism and the citrate cycle (TCA cycle).

**Figure 5:**
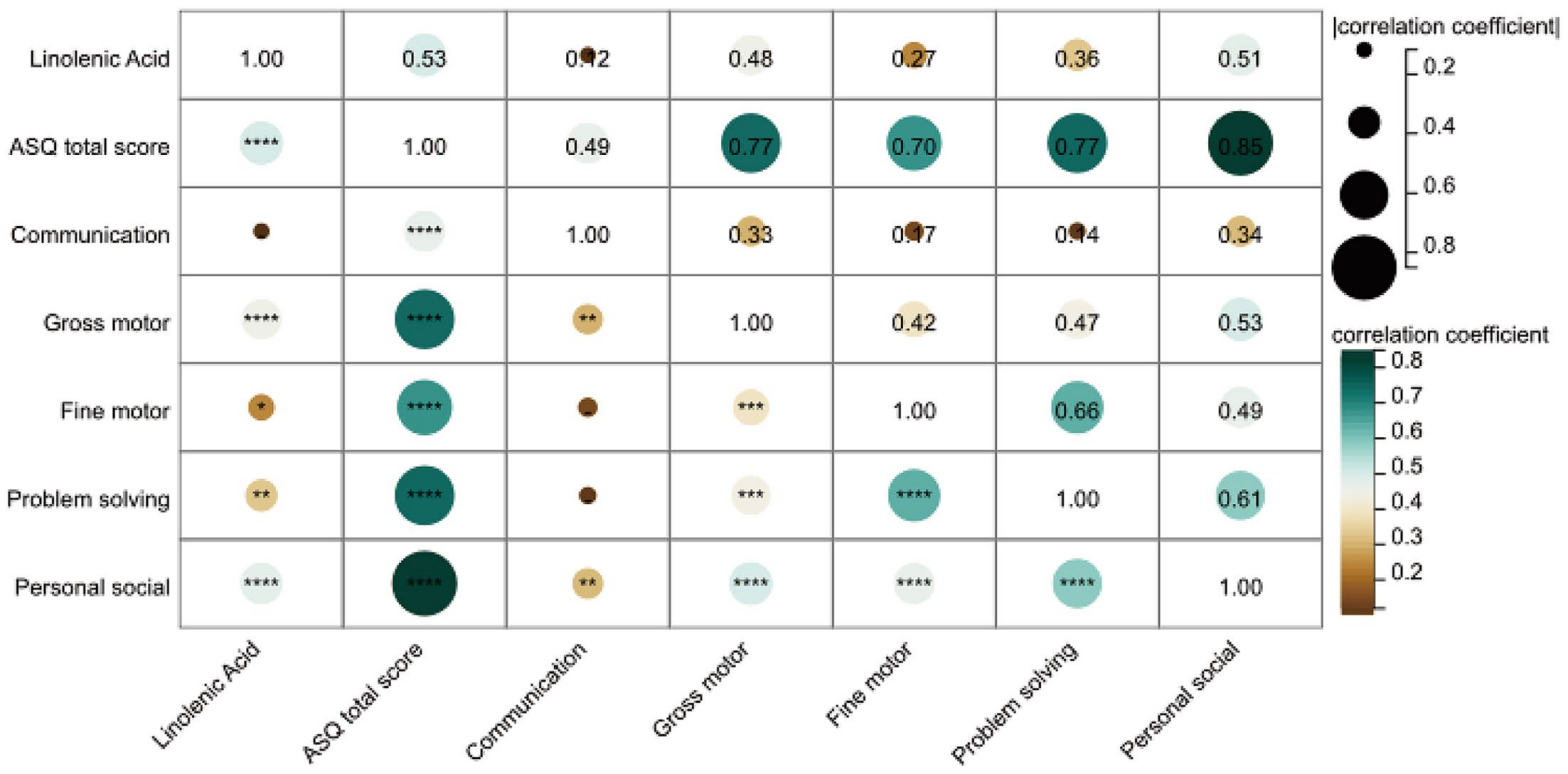
Heatmap representation of Spearman correlation between ASQ-3 scores and linoleic acid.

Next, we analyzed the correlation between linolenic acid and the five domains of the ASQ-3 score. As shown in Figure 5, spearman correlation analysis revealed that linolenic acid was positively correlated with gross motor, fine motor, problem-solving, and personal-social skills, and strongly positively correlated with the ASQ total score (*r* = 0.53, *p* < 0.0001). After adjusting for age at surgery, gender, BMI *z*-score, and SpO_2_, linolenic acid remained positively correlated with the ASQ total score, gross motor skills, and personal-social skills (Supplementary Table S7).

### Bacteria and metabolites as biomarkers for neurodevelopmental disorders

**Figure 6:**
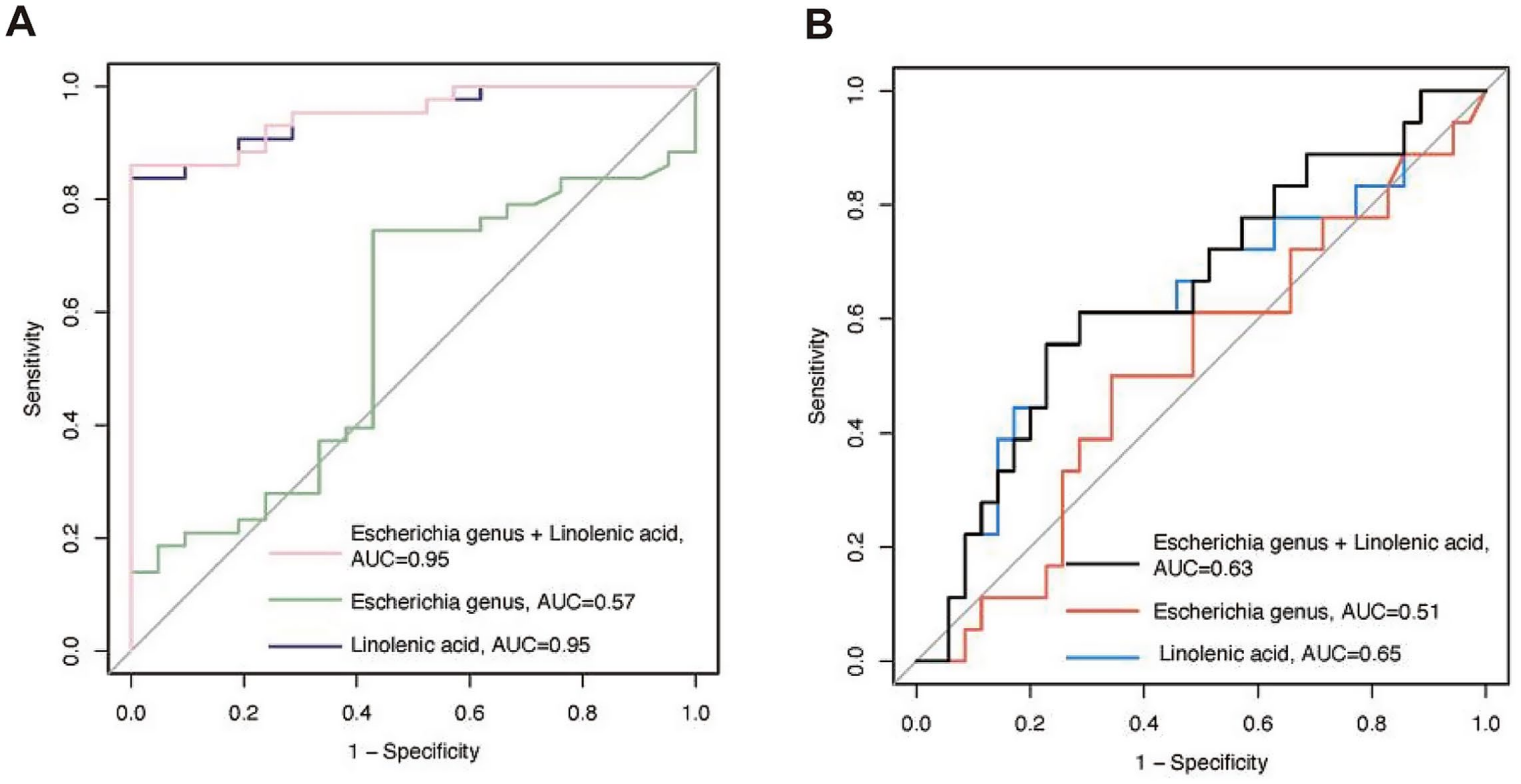
The receiver operating characteristic (ROC) curves for predicting the probability of neurodevelopmental disorders (ND) using bacteria and linoleic acid. **(A)** Predicting the probability of ND at admission based on bacteria and metabolites. **(B)** Predicting the probability of ND during the follow-up period based on bacteria and metabolites collected at admission. AUC, area under the curve.

Finally, we calculated the sensitivity and specificity of the Escherichia genus, linolenic acid, and their combination in identifying ND using logistic regression analysis. The areas under the ROC curve (AUC) were 0.57, 0.95, and 0.95, respectively, indicating that the serum metabolite linolenic acid had higher predictive accuracy for ND (Figure 6A).

### Follow-up cohort

We additionally conducted longitudinal tracking of neurodevelopmental outcomes. The follow-up period was 21.24 (3.74) months, and neurodevelopmental assessments were performed using an online questionnaire. A total of 53 patients completed the questionnaire. Patients who completed the questionnaire had shorter hospital stays compared to those who did not [25.89 (7.31) vs. 32.55 (10.09) days, *p* = 0.012]. Follow-up age, gender, CPB time, ACC time, LOS in ICU and hospital, and ASQ scores were comparable between those who completed the questionnaire and those who did not. Similarly, ROC curve analysis showed that the predictive ability of the Escherichia genus, linolenic acid, and their combination for long-term postoperative neurodevelopmental disorders decreased, with AUC values of 0.51, 0.63, and 0.65, respectively (Figure 6B).

## Discussion

Few clinical studies have investigated changes in gut microbiota and serum metabolomics during the neurodevelopment of CHD. In this prospective study, we are the first to report alterations in gut microbiota and serum metabolomics in children with ND in CHD population. Although no significant differences were observed in gut microbiota between the ND and NND groups at the overall level (alpha and beta diversity), differences emerged at the genus level. The NND group exhibited a higher abundance of the Hungatella genus, while the Bacillus and Escherichia genera were enriched in the ND group. These bacteria may participate in normal neurodevelopment and disease states through their metabolites and immunomodulatory effects. Additionally, metabolomics revealed that a reduction in linolenic acid is a key metabolite associated with neurodevelopmental delays, consistent with previous research findings (Ahrens et al., 2024). This discovery is crucial for interventions targeting ND in CHD.

### Chronic hypoxia affects neurodevelopment and gut microbiota

In this study, we are the first to report the coexistence of ND with changes in bacteria and metabolites in CHD patients, while also examining the roles of individual characteristics and social factors. First, after controlling for potential neurodevelopmental influencing factors such as age, gender, BMI *z*-score, prematurity, and maternal education level, multivariate logistic regression analysis indicated that SpO_2_ level remains a protective factor against ND (OR = 0.89, 95% CI: 0.80–0.97, *p* = 0.04). Polynomial regression analysis further revealed a positive correlation between SpO_2_ level and ASQ total scores (*r*^2^ = 0.10, *p* = 0.01). Additionally, based on PERMANOVA results, we found that SpO_2_ level is one of the key factors affecting microbial structure (*r*^2^ = 0.05, *p* < 0.05). However, SpO_2_ level did not explain the differences in metabolomics (*r*^2^ = 0.017, *p* > 0.05).

CHD, particularly CCHD, leads to chronic fetal hypoxia in utero. Multimodal fetal imaging has detected a 10% reduction in ascending aortic SpO_2_, resulting in a 15% decrease in cerebral oxygen delivery and a 32% reduction in oxygen consumption (Sun et al., 2015). Neuroimaging studies indicate that CCHD fetuses exhibit cortical developmental changes and reduced brain volume, correlating with fetal cerebral oxygen consumption (Sun et al., 2015; Clouchoux et al., 2013). Animal models confirm that chronic hypoxia causes abnormal brain development, including altered brain morphology, reduced brain weight, impaired oligodendrocyte maturation, inadequate myelination, and microglial activation (Cai et al., 2024). The impact of chronic hypoxia on neurodevelopmental outcomes is likely multifactorial, involving hypoxia-induced effects on brain structure and microstructure, as well as medical, social, and environmental factors associated with severe cyanosis (Sood et al., 2024). Hypoxia-induced microbial changes can affect central nervous system activity through immune, endocrine, and vagal pathways. Proof-of-concept studies show that microbial alterations lead to varied behavioral changes in animals, including motor, learning, and memory deficits. However, it remains unclear whether microbiota is altered by physiologically relevant risk factors for ND or whether specific microbial species are causally linked to ND. This study suggests that ND results from the synergistic effects of environmental factors and gut microbiota. Further research is needed to elucidate the molecular basis of interactions between genetic and environmental risk factors.

### Gut-brain axis

The colonization of gut microbiota coincides with the dynamic phase of postnatal brain development. In this study, the average age of CHD patients at admission was 6.44 ± 2.65 months. The first year of life is a critical period for the *de novo* assembly of the complex microbial ecosystem (Schmidt et al., 2018; Shi et al., 2025). Recent studies have shown that changes in gut microbiota composition at 6 months of age are significantly associated with infants’ language, motor, and cognitive scores (Cerdo et al., 2023). Animal models have revealed that gut microbiota influences social behavior and cognitive function (Mao et al., 2020). We found that Firmicutes positively correlated with ASQ total scores and gross motor skills, while Proteobacteria showed a negative correlation with personal-social skills; Escherichia genus was negatively correlated with gross motor skills. Those findings not entirely consistent with Manderino’s et al. (2017) study, who found that Firmicutes were positively correlated with immediate memory, and Proteobacteria were negatively correlated with memory and language. However, both studies emphasize the positive relationship between Firmicutes and neurodevelopment outcomes, and a negative correlation between Proteobacteria and neurodevelopmental outcomes.

### The role of metabolomics in neurodevelopment

In our cohort study, neurodevelopmental disorders were associated with alterations in various metabolites, with the most significant being linolenic acid. Linolenic acid is a polyunsaturated fatty acid (PUFA) belonging to the omega-3 fatty acid family, primarily existing in two forms: ALA (alpha-linolenic acid) and GLA (gamma-linolenic acid). The biological functions of linolenic acid include participation in cell membrane structure and the generation of anti-inflammatory mediators such as prostaglandins and leukotrienes through metabolism (Bazinet and Laye, 2014). It also reduces blood lipids and decreases the risk of atherosclerosis. Furthermore, linolenic acid, particularly ALA, is crucial for the development of the nervous system, a finding supported by our research. A randomized controlled clinical trial revealed that supplementation with omega-3 and omega-6 fatty acids can improve cognitive dysfunction (Stavrinou et al., 2020). However, Vered et al. (2025) suggested that linolenic acid may increase executive dysfunction. The relationship between metabolites and the nervous system is complex and even contradictory, as seen not only with specific metabolites like linolenic acid but also with SCFAs. For example, acetic acid and propionic acid can improve the ultrastructure of hippocampal synapses and promote microglial maturation, thereby enhancing cognitive function. However, the effects of SCFAs are not always positive. Studies have shown that propionic acid is associated with oxidative stress, astrogliosis, hyperactivity, and social behavior abnormalities in the central nervous system of ASD rat models (Shultz et al., 2008). *In vivo* models of Parkinson’s disease have demonstrated that SCFAs promote the aggregation of α-synuclein in specific brain regions and exacerbate motor deficits (Sampson et al., 2016). In Alzheimer’s disease models, SCFAs can increase amyloid-beta deposition and microglial-derived ApoE expression (Colombo et al., 2021). Metabolites exert neuroprotective or neurotoxic effects in preclinical *in vivo* and *in vitro* models; however, convincing clinical studies are still lacking. When evaluating the overall impact of specific metabolites on host health, it is particularly important to consider genetic background, disease state, and various confounding factors. In summary, it is reasonable to believe that neurotransmitter-like substances and active mediators produced by gut microbiota or metabolites send signals to the brain via the peripheral nervous system, influencing and participating in the activities of the nervous system.

This study is the first to reveal a significant association between linolenic acid levels and neurodevelopment in children with CHD. We found that serum linolenic acid levels were significantly lower in the ND group compared to the NND group (*p* < 0.0001), and showed a strong positive correlation with overall neurodevelopmental scores (ASQ total score, *r* = 0.53, *p* < 0.0001). Significant positive correlations were also observed between linolenic acid levels and specific functional domains, including gross motor, fine motor, problem-solving, and personal-social skills. These findings suggest that serum linolenic acid levels may serve as an early warning indicator for neurodevelopmental risk in CHD patients, and that monitoring and supplementation during critical neurodevelopmental periods could potentially improve motor and social deficits.

While this study establishes for the first time the clinical association between linolenic acid levels and neurodevelopment in CHD children, the underlying mechanisms require further validation through animal models and cellular experiments, which will be the focus of our future research.

### Confounding factors affecting neurodevelopment and microbiota

With the establishment of a long-term follow-up cohort for brain development in CHD, it has been observed that ND persist even after surgical intervention has relieved hypoxia. This suggests that neurodevelopmental outcomes may be the result of a combination of multiple factors, including genetic, social, familial, and perioperative risk factors (Bittinger et al., 2020; Delgado et al., 2024). In our follow-up cohort, we retrospectively compared the surgical information between the ND and NND groups. We found no significant differences in CPB time, ACC time, or surgical complexity (RACHS-1 score) between the ND and NND groups (all of *p*-values >0.05). Furthermore, studies indicate that perioperative factors can only explain 5 to 8% of the variability in neurodevelopmental outcomes (International Cardiac Collaborative on Neurodevelopment Investigators, 2016), while 33% is determined by innate patient- and family-related variables (McCusker et al., 2010). Therefore, at least in our study, perioperative factor does not appear to be significant determinants of neurodevelopmental outcomes.

In the preoperative neurodevelopmental assessment cohort, there was a difference in maternal education level between the ND and NND groups, with lower maternal education level in the ND group compared to NND group (*p* = 0.02). This finding is consistent with previous research, where Bucholz et al. (2021) discovered that lower maternal education is associated with delays in communication and problem-solving skills in children with CHD by age 3, as well as delays in fine motor skills between ages 3 and 5 (Davey et al., 2021). In the follow-up cohort, although the trend of lower maternal education levels in the ND group compared to the NND group persisted, no significant difference was observed (*p* = 0.91), possibly due to the limited sample size affecting the results. Furthermore, the socioeconomic status of the family has a significant impact on neurodevelopment, with evidence suggesting a relationship between levels of poverty and brain structure and function (Dumornay et al., 2023). Unfortunately, in this study, we were unable to collect data on the economic income of the patients’ families, but we plan to conduct more comprehensive cohort studies in the future. In summary, we believe that the effects of social and familial factors on children’s neurodevelopment are difficult to explain and control, necessitating large-sample cohort studies to manage various confounding factors.

### Epidemiology

Currently, number of studies aiming to improve the diagnosis, prognosis, and treatment options for ND in CHD. For instance, prenatal diagnosis of CHD can lower the risk of ND by optimizing perinatal care, while implementing personalized developmental care in intensive care units can promote better neurodevelopmental outcomes. To understand the complex interplay of genetic, environmental, and social factors that contribute to the biological basis of ND, it is crucial to consider the gut microbiome. The microbiome serves as an important interface between host genetics and environmental exposures and is an essential regulator of nutrition, immunity, metabolism, and behavior. Davari et al. (2013) found that probiotic supplementation could restore long-term potentiation (LTP) function in the hippocampus of mice. LTP is an activity-dependent change in synaptic efficacy considered a cellular mechanism for learning and memory. Savignac et al. (2015) discovered that Bifidobacterium positively affects stress-related changes, cognitive processes, and emotional behavior through GABAA receptors.

Given the growing evidence that the gut microbiome is a key regulator of neurodevelopment, we believe that microbiome-based strategies could serve as potential therapeutic methods to improve ND in CHD populations. Currently, there are no effective treatments specifically targeting ND in CHD, highlighting the significant potential of microbiome-based therapies as pioneering treatments in this field. However, challenges remain due to the multifaceted nature of gut microbiota in neurodevelopment, making it difficult to identify key bacteria that influence specific neurodevelopmental phenotypes, which complicates subsequent intervention strategies.

To date, there has been extensive research on the effects of microbial interventions in humans and animals, yet their efficacy remains a topic of debate. It is essential to consider several factors when evaluating the effectiveness of these microbial treatments: first, the number of clinical samples, as this directly impacts the reliability of the research results; second, the duration of the study, which should consider the long-term neurobiological effects of these microbial treatments; and finally, when conducting comparative analyses of various phenotypes, it is best to include changes in the microbiome.

### Limitations

In this study, we explored the relationship between gut microbiota, serum metabolites, and neurodevelopmental characteristics in CHD. However, the 16S rRNA sequencing we used only provides compositional information about bacteria, lacking functional insights. Additionally, in this study, the age was range from 1 to 11 months, which includes both neonatal and infant stages, means that factors such as age progression and dietary changes can influence gut microbiota composition (Stewart et al., 2018). This adds complexity in establishing links and causal effects between specific phenotypes during infancy and gut microbiota. The biases were minimized by controlling for factors known to affect neurodevelopment and the gut microbiota. Future research should involve large cohort studies with more precise control over these potential variables. Overall, whether the findings of this study are generalizable across all variations of hypoxia regimens and neurodevelopmental paradigms in humans needs further investigation.

## Conclusion

In summary, the understanding and treatment of ND in CHD populations are still in their early stages. Regarding this study, we found that the influence of metabolites on CHD-related ND is likely greater than that of gut microbiota, with linolenic acid playing a crucial role in neurodevelopment. This provides new experimental evidence for reducing the risk of ND in CHD population in the future. However, it is important to note that ND is a multifactorial condition influenced by the complex interplay of genetic and environmental factors. Further standardized and comprehensive large-sample studies are needed to better understand these connections.

## Data Availability

The original contributions presented in the study are publicly available. This data can be found in here: https://ngdc.cncb.ac.cn/gsa/, accession number PRJCA043554.
