# Supplementary material for: Association between neurodevelopmental disorders in congenital heart disease and changes in circulatory metabolites and gut microbiota composition
Source: Front Microbiol. 2025 Jul 30;16:1639057. doi: 10.3389/fmicb.2025.1639057 (PMC12343688; doi:10.3389/fmicb.2025.1639057)
Supplement: Supplementary file 1 [file Supplementary_file_1.docx]

| Table S1. Identifying risk factors for neurodevelopmental disorders based on multivariate logistic regression method. | | | |
| --- | --- | --- | --- |
| Variables | Z value | OR (95%CI) | P value |
| Age at surgery | 0.31 | 1.03 (0.82, 1.30) | 0.75 |
| Gender | -2.02 | 1.73 (0.55, 5.54) | 0.34 |
| BMI z score | 0.94 | 1.08 (0.82, 1.45) | 0.58 |
| Premature | 0.54 | 0.51 (0.82, 1.45) | 0.42 |
| SpO_2_ | -0.78 | 0.89 (0.80, 9.97) | 0.04 |
| Maternal education | -1.70 | 0.38 (0.11, 1.09) | 0.08 |
| BMI, body mass index.   \| Table S2. Analyzing the association between clinical characteristics and ASQ scores based on linear regression method. \| \| \| \| \| \| \| --- \| --- \| --- \| --- \| --- \| --- \| \|  \| Variables \| β (95%CI) \| P value \| R^2^ adjusted \| P value model fit \| \| ASQ total score \|  \|  \|  \|  \|  \| \|  \| Age at surgery \| -1.10 (-4.70 to 2.50) \| 0.54 \| 0.22 \| 0.001 \| \|  \| Gender \| 7.01 (-11.67 to 25.69) \| 0.45 \|  \|  \| \|  \| BMI z score \| -0.75 (-5.07 to 3.57) \| 0.72 \|  \|  \| \|  \| Premature \| 7.57 (-17.39 to 32.54) \| 0.54 \|  \|  \| \|  \| SpO_2_ \| 2.04 (0.61 to 3.46) \| 0.01 \|  \|  \| \|  \| Maternal education \| 21.98 (5.59 to 38.36) \| 0.01 \|  \|  \| \| Communication \|  \|  \|  \|  \|  \| \|  \| Age at surgery \| 0.08 (-0.84 to 1.01) \| 0.85 \| 0.11 \| 0.04 \| \|  \| Gender \| 0.82 (-3.96 to 5.62) \| 0.73 \|  \|  \| \|  \| BMI z score \| 0.08 (-1.02 to 1.19) \| 0.87 \|  \|  \| \|  \| Premature \| 4.96 (-1.44 to 11.36) \| 0.12 \|  \|  \| \|  \| SpO_2_ \| 0.45 (0.09 to 0.82) \| 0.02 \|  \|  \| \|  \| Maternal education \| 2.98 (-1.21 to 7.19) \| 0.16 \|  \|  \| \| Gross motor \|  \|  \|  \| 0.34 \| <0.001 \| \|  \| Age at surgery \| -0.63 (-1.48 to 0.21) \| 0.14 \|  \|  \| \|  \| Gender \| 1.47 (-2.90 to 5.86) \| 0.50 \|  \|  \| \|  \| BMI z score \| -0.45 (-1.46 to 0.55) \| 0.37 \|  \|  \| \|  \| Premature \| 4.20 (-1.65 to 10.05) \| 0.15 \|  \|  \| \|  \| SpO_2_ \| 0.84 (0.50 to 1.17) \| <0.001 \|  \|  \| \|  \| Maternal education \| 1.65 (-2.18 to 5.50) \| 0.39 \|  \|  \| \| Fine motor \|  \|  \|  \|  \|  \| \|  \| Age at surgery \| 0.09 (-0.84 to 1.04) \| 0.83 \| 0.12 \| 0.03 \| \|  \| Gender \| 4.05 (-0.84 to 8.95) \| 0.10 \|  \|  \| \|  \| BMI z score \| 0.09 (-1.03 to 1.23) \| 0.86 \|  \|  \| \|  \| Premature \| -1.06 (-7.61 to 5.48) \| 0.75 \|  \|  \| \|  \| SpO_2_ \| 0.29 (-0.07 to 0.67) \| 0.12 \|  \|  \| \|  \| Maternal education \| 5.98 (1.68 to 10.28) \| 0.01 \|  \|  \| \| Problem solving \|  \|  \|  \|  \|  \| \|  \| Age at surgery \| -0.74 (-1.79 to 0.29) \| 0.15 \| 0.09 \| 0.06 \| \|  \| Gender \| -2.62 (-8.04 to 2.79) \| 0.33 \|  \|  \| \|  \| BMI z score \| -0.31 (-1.56 to 0.94) \| 0.62 \|  \|  \| \|  \| Premature \| -1.98 (-9.22 to 5.26) \| 0.59 \|  \|  \| \|  \| SpO_2_ \| 0.10 (-0.30 to 0.52) \| 0.61 \|  \|  \| \|  \| Maternal education \| 4.80 (0.04 to 9.55) \| 0.04 \|  \|  \| \| Personal-social \|  \|  \|  \|  \|  \| \|  \| Age at surgery \| 0.09 (-1.13 to 1.32) \| 0.87 \| 0.07 \| 0.09 \| \|  \| Gender \| 3.27 (-3.09 to 9.65) \| 0.30 \|  \|  \| \|  \| BMI z score \| -0.16 (-1.64 to 1.31) \| 0.82 \|  \|  \| \|  \| Premature \| 1.45 (-7.06 to 9.97) \| 0.73 \|  \|  \| \|  \| SpO_2_ \| 0.33 (-0.15 to 0.82) \| 0.17 \|  \|  \| \|  \| Maternal education \| 6.54 (0.95 to 12.13) \| 0.02 \|  \|  \| \| ASQ, ages and stages questionnaire; BMI, body mass index. \| \| \| \| \| \| | | | |

| Table S3. Random forest model calculates the bacteria most important to differences. | | |
| --- | --- | --- |
| Bacteria | Importance |  |
| g__Escherichia | 2.8257 |  |
| g__Klebsiella | 2.37882 |  |
| g__Morganella | 2.24139 |  |
| g__Citrobacter | 2.19246 |  |
| g__Bilophila | 1.85546 |  |
| g__Proteus | 1.82675 |  |
| g__Enterobacter | 1.59953 |  |
| g__Bacteroides | 1.43565 |  |
| g__unclassified | 1.41838 |  |
| g__unclassified_f__Peptostreptococcaceae | 1.41705 |  |
| g__Corynebacterium | 1.37846 |  |
| g__Streptococcus | 1.35185 |  |
| g__Coprobacillus | 1.3063 |  |
| g__Mediterraneibacter | 1.28482 |  |
| g__Rothia | 1.10013 |  |
| g__Peptoniphilus | 1.001 |  |
| g__Aggregatibacter | 1.001 |  |
| g__Faecalibacterium | 0.79987 |  |
| g__Limosilactobacillus | 0.66573 |  |
| g__Finegoldia | 0.53201 |  |
| g__Catonella | 0.38117 |  |
| g__Serratia | 0.38098 |  |
| g__Hungatella | 0.20842 |  |
| g__Bradyrhizobium | 0.20113 |  |
| g__Caecibacterium | 0.15528 |  |
| g__Staphylococcus | 0.14132 |  |
| g__Acinetobacter | 0.03673 |  |
| g__Enterococcus | 0.01955 |  |
| g__Dermabacter | 0 |  |
| g__Frisingicoccus | 0 |  |
| g__Cedecea | 0 |  |
| g__unclassified_f__Tissierellaceae | 0 |  |
| g__unclassified_f__Coriobacteriaceae | 0 |  |
| g__Gardnerella | 0 |  |
| g__unclassified_o__Burkholderiales | 0 |  |
| g__Providencia | 0 |  |
| g__Acidovorax | 0 |  |
| g__Massilioclostridium | 0 |  |
| g__Megamonas | 0 |  |
| g__Sphingobium | 0 |  |
| g__Atopobium | 0 |  |
| g__Paenibacillus | 0 |  |
| g__Veillonella | 0 |  |
| g__Eikenella | 0 |  |
| g__Fusobacterium | 0 |  |
| g__Collinsella | 0 |  |
| g__Parvimonas | 0 |  |
| g__unclassified_f__Oscillospiraceae | 0 |  |
| g__unclassified_o__Eubacteriales | 0 |  |
| g__Anaerotignum | 0 |  |
| g__Bosea | 0 |  |
| g__Shuttleworthia | 0 |  |
| g__Peptostreptococcus | 0 |  |
| g__unclassified_p__Firmicutes | 0 |  |
| g__Pseudomonas | 0 |  |
| g__Stenotrophomonas | 0 |  |
| g__Weissella | 0 |  |
| g__unclassified_f__Lachnospiraceae | 0 |  |
| g__unclassified_f__Erysipelotrichaceae | 0 |  |
| g__Edwardsiella | 0 |  |
| g__Alistipes | 0 |  |
| g__Enhydrobacter | 0 |  |
| g__unclassified_c__Betaproteobacteria | 0 |  |
| g__Buttiauxella | 0 |  |
| g__Haemophilus | 0 |  |
| g__Eisenbergiella | 0 |  |
| g__Ralstonia | 0 |  |
| g__Flavonifractor | -0.06526 |  |
| g__Anaerococcus | -0.07189 |  |
| g__Paracoccus | -0.11967 |  |
| g__Bifidobacterium | -0.20041 |  |
| g__Lactococcus | -0.26769 |  |
| g__Gemella | -0.31003 |  |
| g__Lacticaseibacillus | -0.32419 |  |
| g__unclassified_d__unclassified | -0.50544 |  |
| g__Ligilactobacillus | -0.51811 |  |
| g__Campylobacter | -0.62462 |  |
| g__Erysipelatoclostridium | -0.63148 |  |
| g__Novosphingobium | -0.64446 |  |
| g__Lachnoclostridium | -0.72546 |  |
| g__unclassified_d__Bacteria | -0.78496 |  |
| g__Clostridium | -0.84097 |  |
| g__Neisseria | -0.97381 |  |
| g__Tropheryma | -1 |  |
| g__Gleimia | -1.001 |  |
| g__Sphingomonas | -1.01193 |  |
| g__Clostridioides | -1.02134 |  |
| g__unclassified_f__Enterobacteriaceae | -1.09471 |  |
| g__Bacillus | -1.10487 |  |
| g__Prevotella | -1.15063 |  |
| g__unclassified_f__Micrococcaceae | -1.28319 |  |
| g__Granulicatella | -1.34695 |  |
| g__Butyricicoccus | -1.41214 |  |
| g__Blautia | -1.54635 |  |
| g__unclassified_f__Oxalobacteraceae | -1.68783 |  |
| g__unclassified_o__Lactobacillales | -1.7172 |  |
| g__unclassified_f__Streptococcaceae | -1.82944 |  |
| g__Anaerostipes | -1.92626 |  |
| g__Eggerthella | -2.11827 |  |
| g__Burkholderia | -2.70841 |  |
| g__Actinomyces | -2.90374 |  |
| g__Lactobacillus | -2.92398 |  |

| Table S4. The effect of characteristics on differences in gut microbial variation based on permutational multivariate analysis of variance method. | | | | |
| --- | --- | --- | --- | --- |
| Characteristics | F | r^2^ | P value | P adjust value |
| Age at surgery | 3.55 | 0.05 | 0.002 | 0.01 |
| SpO_2_ | 3.23 | 0.05 | 0.003 | 0.01 |
| Breastfeeding | 1.50 | 0.02 | 0.15 | 0.30 |
| Born mode | 1.13 | 0.02 | 0.30 | 0.45 |
| Gender | 0.71 | 0.01 | 0.69 | 0.82 |
| Premature | 0.52 | 0.01 | 0.88 | 0.88 |
| BMI z score | 0.71 | 0.01 | 0.68 | 0.81 |
| BMI, body mass index. | | | | |

| Table S5. The differential metabolites between ND and NND groups (unadjusted). | | | |
| --- | --- | --- | --- |
| Metabolites | FC | log2(FC) | P value |
| 3-Hydroxy-3-methylglutarylcarnitine | 1.2095 | 0.27447 | 0.00010532 |
| metab_3145 | 1.3009 | 0.37949 | 0.00010568 |
| metab_7993 | 0.95992 | -0.059021 | 0.00011573 |
| metab_10723 | 0.9249 | -0.11263 | 0.00013332 |
| metab_2677 | 0.84577 | -0.24167 | 0.00015293 |
| metab_12193 | 1.6954 | 0.76159 | 0.00015563 |
| metab_8043 | 0.86862 | -0.20321 | 0.0001602 |
| metab_12188 | 1.5887 | 0.66788 | 0.00020731 |
| metab_11697 | 0.84241 | -0.24741 | 0.00020968 |
| metab_11437 | 1.1819 | 0.24108 | 0.0002188 |
| metab_8830 | 1.8417 | 0.88104 | 0.00022356 |
| metab_5193 | 0.94308 | -0.084546 | 0.0002239 |
| metab_13059 | 1.2119 | 0.27731 | 0.00027814 |
| metab_17563 | 0.89427 | -0.16121 | 0.00028106 |
| metab_8378 | 1.5423 | 0.62505 | 0.00030333 |
| metab_17691 | 0.96454 | -0.052083 | 0.00030463 |
| metab_11250 | 1.5597 | 0.64131 | 0.00030819 |
| metab_8042 | 0.88441 | -0.17722 | 0.00033249 |
| metab_1298 | 1.09 | 0.12435 | 0.00034245 |
| Acotiamide | 1.4114 | 0.49716 | 0.00035871 |
| metab_14611 | 0.71611 | -0.48176 | 0.00039794 |
| metab_3107 | 1.0155 | 0.022204 | 0.00041372 |
| metab_17463 | 1.1194 | 0.16276 | 0.00041821 |
| metab_17256 | 1.2133 | 0.27892 | 0.00042921 |
| metab_10917 | 0.94491 | -0.081756 | 0.00043055 |
| metab_13947 | 1.2824 | 0.3588 | 0.00044346 |
| metab_9833 | 0.98725 | -0.018509 | 0.00044997 |
| metab_1261 | 1.1774 | 0.23565 | 0.00048289 |
| metab_2202 | 1.2592 | 0.3325 | 0.00049293 |
| metab_110 | 0.89533 | -0.15952 | 0.00053271 |
| Pipecolic Acid | 1.0361 | 0.051169 | 0.00056078 |
| metab_9657 | 1.2726 | 0.34777 | 0.0005689 |
| metab_9707 | 0.88716 | -0.17274 | 0.00060897 |
| 3-Fucosyllactose | 1.5933 | 0.67202 | 0.00064357 |
| Gibberellin A37 | 1.73 | 0.79079 | 0.00066203 |
| metab_11515 | 1.3291 | 0.41049 | 0.00068951 |
| metab_12025 | 1.0964 | 0.13283 | 0.00069507 |
| Phenyl glucuronide | 1.4756 | 0.56131 | 0.00070057 |
| metab_386 | 2.0792 | 1.056 | 0.00071157 |
| metab_14156 | 0.94175 | -0.086583 | 0.0007316 |
| Antibiotic LL-AB 664 | 1.5551 | 0.63702 | 0.00075838 |
| metab_14176 | 0.92984 | -0.10494 | 0.00079006 |
| metab_13780 | 0.95396 | -0.067993 | 0.00079484 |
| metab_11278 | 0.93397 | -0.098547 | 0.0008389 |
| metab_11371 | 1.1513 | 0.20326 | 0.00083989 |
| metab_2303 | 0.87428 | -0.19383 | 0.00084689 |
| Milbemycin alpha6 | 1.1907 | 0.25181 | 0.00088094 |
| metab_10428 | 0.89783 | -0.15549 | 0.00091294 |
| metab_2684 | 0.84769 | -0.23838 | 0.00094554 |
| metab_9838 | 1.0478 | 0.06738 | 0.00094669 |
| metab_11357 | 1.1115 | 0.15249 | 0.00095291 |
| metab_7128 | 1.0361 | 0.051143 | 0.00095978 |
| metab_3799 | 1.1277 | 0.17343 | 0.00097951 |
| metab_11197 | 1.8625 | 0.89725 | 0.0010147 |
| metab_12160 | 1.2191 | 0.28587 | 0.001038 |
| 2-benzyl-N-[(1-methylbenzimidazol-2-yl)methyl]-7-oxoazepane-2-carboxamide | 1.0727 | 0.10127 | 0.0010495 |
| metab_10881 | 0.88753 | -0.17213 | 0.0010532 |
| metab_13581 | 0.95862 | -0.060967 | 0.0010731 |
| metab_8300 | 0.916 | -0.12659 | 0.0010763 |
| metab_2795 | 1.1187 | 0.16181 | 0.0010806 |
| metab_7855 | 1.1581 | 0.21174 | 0.0011033 |
| metab_4387 | 1.0424 | 0.059972 | 0.0011151 |
| DG(22:6(4Z,7Z,10Z,13Z,16Z,19Z)/15:0/0:0) | 1.7166 | 0.77952 | 0.0011168 |
| metab_4834 | 1.2585 | 0.33169 | 0.0011229 |
| metab_4868 | 1.9527 | 0.96549 | 0.0011283 |
| metab_11636 | 1.1026 | 0.14086 | 0.0011378 |
| metab_3710 | 0.96286 | -0.054599 | 0.0011613 |
| metab_10654 | 0.89077 | -0.16688 | 0.0011664 |
| metab_8938 | 1.2743 | 0.34974 | 0.0011704 |
| metab_8934 | 1.1824 | 0.24171 | 0.0011739 |
| Gonal | 0.88436 | -0.17729 | 0.0011965 |
| metab_14268 | 0.9721 | -0.04083 | 0.0012183 |
| metab_14195 | 0.94453 | -0.082335 | 0.0012298 |
| Deoxycorticosterone acetate | 1.0744 | 0.10347 | 0.001232 |
| (-)-Pinocarvone | 1.0519 | 0.073055 | 0.0012527 |
| metab_16392 | 1.1101 | 0.15073 | 0.0012885 |
| L-Theanine | 1.1139 | 0.15565 | 0.0013442 |
| metab_8758 | 0.97573 | -0.035439 | 0.0013603 |
| metab_11895 | 1.0333 | 0.04721 | 0.0013627 |
| metab_10446 | 0.94234 | -0.085685 | 0.0014189 |
| Proglumide | 1.1751 | 0.23283 | 0.0014796 |
| metab_9713 | 1.1908 | 0.25187 | 0.0014888 |
| metab_11446 | 1.2198 | 0.28664 | 0.0014962 |
| Perfluoroheptanoic Acid | 0.87002 | -0.20087 | 0.0015078 |
| metab_17640 | 0.98119 | -0.027402 | 0.0015367 |
| metab_6171 | 1.1271 | 0.17256 | 0.0015578 |
| 2',3'-Dideoxyadenosine-5-triphosphate | 1.9436 | 0.95874 | 0.0015635 |
| N-[(4E,8Z)-1,3-dihydroxyoctadeca-4,8-dien-2-yl]hexadecanamide 1-glucoside | 0.97272 | -0.039902 | 0.0016365 |
| D-Galactose | 1.0476 | 0.067145 | 0.001662 |
| metab_8283 | 0.87786 | -0.18793 | 0.0016654 |
| metab_7554 | 1.7225 | 0.78452 | 0.0016664 |
| metab_13760 | 1.3128 | 0.3926 | 0.0017165 |
| 13-Oxo-9,11-tridecadienoic acid | 1.1284 | 0.17428 | 0.0017314 |
| metab_14476 | 0.94785 | -0.077266 | 0.0017327 |
| 5b-Cholestane-3a,7a,12a,23R,25-pentol | 1.1115 | 0.15249 | 0.0017629 |
| Neocnidilide | 1.0894 | 0.12353 | 0.001783 |
| metab_10053 | 1.2161 | 0.28221 | 0.0018014 |
| metab_1072 | 0.79598 | -0.32919 | 0.0018035 |
| metab_8877 | 0.73398 | -0.44619 | 0.0018088 |
| metab_8259 | 0.93684 | -0.094119 | 0.0018233 |
| N-Succinyl-2-amino-6-ketopimelate | 0.8647 | -0.20973 | 0.0018237 |
| metab_11749 | 1.8475 | 0.88558 | 0.0018323 |
| metab_15536 | 1.1947 | 0.25666 | 0.0018431 |
| metab_7750 | 1.4916 | 0.5769 | 0.0018598 |
| metab_12278 | 1.1321 | 0.17895 | 0.0018655 |
| metab_1251 | 0.93051 | -0.1039 | 0.0018765 |
| Formyl 3-hydroxybutanoate | 1.0408 | 0.057724 | 0.0018901 |
| metab_8003 | 1.434 | 0.52 | 0.001974 |
| metab_4105 | 0.8648 | -0.20956 | 0.0020001 |
| metab_10429 | 0.9135 | -0.13052 | 0.0020077 |
| metab_8065 | 1.1294 | 0.1756 | 0.0020558 |
| metab_304 | 1.1233 | 0.1678 | 0.0020705 |
| metab_14749 | 0.85676 | -0.22303 | 0.0020888 |
| metab_13127 | 1.2316 | 0.30048 | 0.0021094 |
| metab_11290 | 1.5955 | 0.67397 | 0.0021258 |
| metab_15528 | 1.7533 | 0.81003 | 0.0021452 |
| metab_14144 | 0.90776 | -0.13961 | 0.0021467 |
| metab_11445 | 1.2484 | 0.32012 | 0.0021655 |
| metab_16628 | 1.0943 | 0.12996 | 0.0021859 |
| metab_12910 | 0.89888 | -0.1538 | 0.0022284 |
| metab_7904 | 0.96979 | -0.044258 | 0.0022437 |
| metab_492 | 1.2961 | 0.37415 | 0.0022687 |
| 5-Keto-D-Gluconate | 1.1821 | 0.24141 | 0.00227 |
| metab_7464 | 0.95977 | -0.059234 | 0.0022966 |
| metab_10725 | 0.94273 | -0.08508 | 0.0023026 |
| metab_11374 | 1.1887 | 0.24941 | 0.0023112 |
| Kinetin | 1.1686 | 0.22478 | 0.0023353 |
| metab_15470 | 0.95099 | -0.072501 | 0.0023461 |
| metab_5861 | 0.95714 | -0.063201 | 0.0023627 |
| metab_7744 | 1.1113 | 0.15231 | 0.002388 |
| metab_3463 | 1.0717 | 0.099869 | 0.0023913 |
| metab_14952 | 0.92946 | -0.10554 | 0.0024363 |
| 5-Hydroxyindoleacetate | 1.2577 | 0.33074 | 0.0024407 |
| metab_14477 | 0.93548 | -0.096214 | 0.0024473 |
| metab_565 | 1.328 | 0.4092 | 0.0024555 |
| metab_7463 | 0.94581 | -0.080377 | 0.0024591 |
| 12-O-D-Glucuronoside-13-hydroxyoctadec-9Z-enoate | 1.2677 | 0.34225 | 0.0025294 |
| metab_9518 | 0.97884 | -0.030855 | 0.002545 |
| L-Glutamine | 0.98224 | -0.025848 | 0.0025527 |
| 3-Dehydroteasterone | 1.0694 | 0.096752 | 0.0025716 |
| metab_10459 | 0.96485 | -0.051629 | 0.0025752 |
| Emopamil | 1.1509 | 0.20273 | 0.0025882 |
| metab_1013 | 1.3025 | 0.38126 | 0.0025935 |
| metab_3472 | 0.95351 | -0.068681 | 0.0026365 |
| metab_13950 | 1.0992 | 0.13643 | 0.0026451 |
| metab_3143 | 1.0403 | 0.05702 | 0.0026488 |
| Rose oxide (cis) | 1.0906 | 0.12513 | 0.0026618 |
| 4-p-Coumaroylquinic acid | 1.1991 | 0.2619 | 0.002663 |
| metab_246 | 1.1361 | 0.18406 | 0.0026998 |
| metab_4608 | 0.65552 | -0.60928 | 0.0027158 |
| metab_10584 | 0.94084 | -0.087974 | 0.0027503 |
| 2-Quinolinecarboxylic acid | 1.1867 | 0.24697 | 0.0028322 |
| metab_14152 | 1.0969 | 0.1334 | 0.002835 |
| metab_10046 | 1.1223 | 0.16647 | 0.0028385 |
| Cucurbitacin C | 1.3107 | 0.39038 | 0.002884 |
| metab_7435 | 0.95717 | -0.063146 | 0.002892 |
| 6-Hydroxynorketamine | 1.1579 | 0.21153 | 0.0028947 |
| Ubiquinone-1 | 1.144 | 0.19409 | 0.0029438 |
| metab_8248 | 0.96038 | -0.05832 | 0.0030079 |
| metab_11569 | 1.0424 | 0.059865 | 0.0030346 |
| metab_10664 | 1.3941 | 0.47934 | 0.0030925 |
| metab_17453 | 0.92692 | -0.10948 | 0.0031073 |
| metab_12233 | 1.4029 | 0.48845 | 0.0031537 |
| metab_1337 | 1.0441 | 0.062248 | 0.0031756 |
| metab_3239 | 1.0193 | 0.027514 | 0.0032008 |
| L-cis-Cyclo(aspartylphenylalanyl) | 1.5078 | 0.59248 | 0.0032057 |
| Stizolamine | 1.0283 | 0.040299 | 0.0032088 |
| metab_12929 | 0.97754 | -0.032772 | 0.0032432 |
| 10-Hydroperoxy-H4-neuroprostane | 1.1748 | 0.23241 | 0.0032742 |
| metab_7786 | 1.0628 | 0.087803 | 0.0032807 |
| metab_16638 | 1.1759 | 0.23382 | 0.0032815 |
| metab_1203 | 0.94263 | -0.085239 | 0.0032991 |
| metab_8098 | 0.93703 | -0.093835 | 0.0033025 |
| Kutkoside | 0.79334 | -0.33399 | 0.0033248 |
| metab_10641 | 0.97857 | -0.031254 | 0.003327 |
| metab_17582 | 0.96526 | -0.051016 | 0.0033356 |
| metab_1045 | 0.92006 | -0.12019 | 0.0033668 |
| metab_546 | 1.1187 | 0.16185 | 0.0033739 |
| metab_13943 | 1.1882 | 0.24875 | 0.0033793 |
| metab_9627 | 1.1806 | 0.23955 | 0.0033927 |
| metab_14743 | 1.564 | 0.64528 | 0.0033978 |
| metab_2205 | 1.1528 | 0.2051 | 0.0034007 |
| metab_312 | 2.5786 | 1.3666 | 0.0034023 |
| metab_13565 | 0.93023 | -0.10435 | 0.0034464 |
| metab_7860 | 1.036 | 0.051044 | 0.0035188 |
| metab_14567 | 0.91816 | -0.12319 | 0.003531 |
| 16-phenyl tetranor PGF2alpha | 1.2077 | 0.27223 | 0.0035417 |
| metab_3246 | 0.92757 | -0.10848 | 0.0035458 |
| metab_615 | 1.0344 | 0.04879 | 0.0036293 |
| metab_17566 | 0.89898 | -0.15364 | 0.0036321 |
| metab_13463 | 1.2331 | 0.30229 | 0.0036957 |
| metab_10522 | 1.0642 | 0.089816 | 0.0037145 |
| metab_10804 | 1.2747 | 0.35013 | 0.0037174 |
| metab_3790 | 1.118 | 0.16095 | 0.003731 |
| metab_10620 | 0.9346 | -0.097582 | 0.0037414 |
| metab_2741 | 1.0925 | 0.12764 | 0.0037544 |
| metab_8007 | 3.3751 | 1.7549 | 0.0037774 |
| Indoleacetic acid | 1.1914 | 0.25262 | 0.0037777 |
| Naftazone | 0.88257 | -0.18022 | 0.0038134 |
| metab_13065 | 0.91563 | -0.12717 | 0.003827 |
| metab_2982 | 1.1081 | 0.14806 | 0.0038539 |
| metab_13629 | 0.98029 | -0.028721 | 0.0038555 |
| metab_732 | 1.098 | 0.13489 | 0.0038701 |
| metab_13826 | 0.93074 | -0.10355 | 0.003908 |
| metab_10531 | 0.90903 | -0.1376 | 0.0039116 |
| metab_13742 | 1.4246 | 0.51051 | 0.003916 |
| metab_3973 | 1.0338 | 0.047937 | 0.0039372 |
| metab_17475 | 0.8766 | -0.19001 | 0.003951 |
| metab_15385 | 0.95256 | -0.070116 | 0.0039548 |
| 2-(2-(4-Phenoxy-2-propylphenoxy)ethyl)indole-5-acetic acid | 1.7714 | 0.82492 | 0.0039671 |
| metab_17251 | 1.1691 | 0.22537 | 0.0040399 |
| Erythrose | 1.026 | 0.037026 | 0.004045 |
| metab_3037 | 1.1687 | 0.22487 | 0.0040742 |
| Prostaglandin E3 | 1.11 | 0.15057 | 0.004129 |
| metab_12835 | 1.0876 | 0.1212 | 0.0041376 |
| metab_12260 | 0.97297 | -0.03953 | 0.0041383 |
| metab_8273 | 0.90366 | -0.14615 | 0.0041458 |
| metab_14818 | 1.0267 | 0.038038 | 0.0041683 |
| (2S)-2-(4-Chloroanilino)propanoic acid | 0.96363 | -0.053444 | 0.004179 |
| metab_1257 | 1.0481 | 0.067754 | 0.0041913 |
| Eicosopentanoic acid | 1.1534 | 0.20589 | 0.0042611 |
| metab_5786 | 0.91121 | -0.13414 | 0.0042614 |
| metab_5356 | 0.98311 | -0.024572 | 0.004278 |
| metab_9235 | 1.263 | 0.33689 | 0.0042814 |
| metab_2066 | 1.2194 | 0.28615 | 0.0043103 |
| 2-[(4-Hydroxycyclohexyl)amino]-4-(3,6,6-trimethyl-4-oxo-5,7-dihydroindazol-1-yl)benzamide | 1.082 | 0.11373 | 0.0043178 |
| Heptyl 4-hydroxybenzoate | 1.0976 | 0.13441 | 0.0043225 |
| metab_1590 | 0.95038 | -0.073429 | 0.0043325 |
| metab_2824 | 1.0478 | 0.0673 | 0.004336 |
| metab_7923 | 0.98161 | -0.02678 | 0.0044271 |
| metab_11993 | 1.0706 | 0.09846 | 0.0044632 |
| metab_8067 | 1.6162 | 0.69258 | 0.0044908 |
| metab_7280 | 1.0734 | 0.10215 | 0.0044943 |
| metab_3123 | 0.96467 | -0.051893 | 0.0045104 |
| metab_17260 | 1.0537 | 0.075417 | 0.0045863 |
| metab_8452 | 2.0227 | 1.0163 | 0.0046323 |
| metab_8341 | 1.2852 | 0.36196 | 0.0047516 |
| PA(8:0/8:0) | 0.68568 | -0.54439 | 0.0047747 |
| 3-hydroxy-3-methyl-Glutaric acid | 1.0194 | 0.027659 | 0.0047755 |
| metab_3340 | 1.025 | 0.035673 | 0.0047836 |
| metab_10537 | 0.9425 | -0.085434 | 0.00492 |
| metab_7597 | 1.1153 | 0.15739 | 0.004928 |
| metab_14684 | 1.0949 | 0.13082 | 0.0049322 |
| metab_11187 | 1.0783 | 0.10876 | 0.0049352 |
| metab_10267 | 1.146 | 0.1966 | 0.0050327 |
| metab_3111 | 1.0659 | 0.09206 | 0.0050501 |
| metab_16916 | 1.138 | 0.18653 | 0.0050622 |
| Triflumuron | 0.99108 | -0.01293 | 0.0050664 |
| metab_1303 | 1.0343 | 0.048721 | 0.005077 |
| metab_10443 | 1.2112 | 0.27644 | 0.0051259 |
| metab_12788 | 0.93278 | -0.10039 | 0.0051363 |
| Timonacic | 0.91356 | -0.13043 | 0.0051498 |
| metab_10677 | 0.8963 | -0.15795 | 0.0051582 |
| Xanthurenic Acid | 1.1204 | 0.16407 | 0.0051609 |
| S-(3-Oxo-3-carboxy-n-propyl)cysteine | 0.89598 | -0.15846 | 0.0051793 |
| Mukurozidiol | 2.0589 | 1.0419 | 0.0052266 |
| 1,4-Naphthoquinone | 1.3859 | 0.4708 | 0.0052588 |
| metab_17564 | 0.95095 | -0.072557 | 0.0052824 |
| Glutaric Acid | 1.015 | 0.021438 | 0.0053472 |
| metab_7882 | 0.97931 | -0.030155 | 0.0053617 |
| 2-Amino-4-[carbamimidoyl(methyl)amino]butanoic acid | 0.9629 | -0.054537 | 0.0053634 |
| metab_7336 | 1.0368 | 0.052147 | 0.0053949 |
| metab_8749 | 1.2488 | 0.32057 | 0.0054006 |
| metab_13782 | 0.77421 | -0.36921 | 0.0054076 |
| metab_6299 | 0.97735 | -0.033053 | 0.0054164 |
| metab_13959 | 0.48368 | -1.0479 | 0.00543 |
| Beta-D-Glucopyranuronic acid | 0.9892 | -0.01567 | 0.00546 |
| metab_1302 | 1.0967 | 0.13322 | 0.0055023 |
| metab_14574 | 0.83762 | -0.25563 | 0.0055228 |
| metab_12047 | 0.89705 | -0.15674 | 0.0055541 |
| 2,4-Dinitrophenol | 0.96555 | -0.05058 | 0.005559 |
| metab_11283 | 0.87982 | -0.18472 | 0.0056964 |
| Yucalexin P15 | 1.5921 | 0.67092 | 0.0057094 |
| metab_8894 | 1.3283 | 0.40957 | 0.0057227 |
| Taxiphyllin | 1.131 | 0.17762 | 0.0057467 |
| metab_1291 | 0.91953 | -0.12102 | 0.0058115 |
| metab_12449 | 0.97934 | -0.030115 | 0.0058801 |
| metab_12180 | 1.1585 | 0.21221 | 0.0059042 |
| metab_12479 | 0.95848 | -0.061175 | 0.005912 |
| LysoPC(O-18:0/0:0) | 0.95939 | -0.059806 | 0.0059141 |
| metab_3017 | 1.0909 | 0.12547 | 0.0059238 |
| metab_17326 | 1.2155 | 0.28151 | 0.0059493 |
| metab_2387 | 0.65759 | -0.60474 | 0.0059735 |
| FAHFA(16:0/11-O-16:0) | 1.0949 | 0.13077 | 0.0059753 |
| metab_2226 | 0.92469 | -0.11295 | 0.0060072 |
| metab_12024 | 1.8849 | 0.91453 | 0.0060247 |
| metab_2939 | 1.165 | 0.22027 | 0.0060249 |
| Mupirocin | 1.0824 | 0.11427 | 0.0060326 |
| metab_15271 | 0.92103 | -0.11868 | 0.0060603 |
| metab_4535 | 0.8934 | -0.16263 | 0.0060705 |
| metab_14647 | 1.0544 | 0.076433 | 0.0060774 |
| metab_8651 | 1.0427 | 0.060288 | 0.0061254 |
| metab_7413 | 1.1144 | 0.15621 | 0.0061365 |
| metab_12850 | 0.94914 | -0.075305 | 0.0061372 |
| metab_2951 | 1.1334 | 0.1806 | 0.0061834 |
| metab_13640 | 0.95506 | -0.066333 | 0.0061927 |
| metab_10146 | 1.4689 | 0.55475 | 0.0062042 |
| metab_11611 | 1.0414 | 0.058525 | 0.0062255 |
| metab_6338 | 0.84144 | -0.24907 | 0.0062404 |
| Sobetirome | 1.3468 | 0.4295 | 0.0062449 |
| metab_10515 | 0.96783 | -0.047177 | 0.0063385 |
| metab_3253 | 1.1383 | 0.18692 | 0.0063779 |
| metab_1953 | 1.0409 | 0.05783 | 0.0063782 |
| metab_12338 | 1.7114 | 0.77522 | 0.0064317 |
| metab_17552 | 1.0528 | 0.07423 | 0.0064998 |
| metab_7015 | 1.0211 | 0.030158 | 0.0065226 |
| metab_12212 | 1.0748 | 0.10402 | 0.0065791 |
| 4-Quinolinecarboxylic Acid | 3.6938 | 1.8851 | 0.0066092 |
| 7-[(1R,2R,3R,5S)-3,5-Dihydroxy-2-[(3S)-3-hydroxy-5-phenylpent-1-enyl]cyclopentyl]-N-ethylhept-5-enamide | 1.1014 | 0.13939 | 0.0066222 |
| metab_14658 | 0.97746 | -0.03289 | 0.0066914 |
| metab_16492 | 1.1268 | 0.17217 | 0.0067072 |
| metab_2548 | 0.83813 | -0.25475 | 0.0067105 |
| metab_9179 | 1.2611 | 0.33467 | 0.0068338 |
| metab_4609 | 0.87359 | -0.19498 | 0.0068372 |
| metab_7249 | 1.207 | 0.27143 | 0.0069209 |
| metab_2891 | 1.2152 | 0.28117 | 0.0069244 |
| metab_15192 | 0.95426 | -0.067546 | 0.0070211 |
| Hypusine | 2.2901 | 1.1954 | 0.0070269 |
| metab_661 | 1.0285 | 0.040528 | 0.0071663 |
| metab_10290 | 1.0575 | 0.080677 | 0.0071749 |
| metab_7589 | 1.3374 | 0.41947 | 0.0072203 |
| metab_16903 | 0.75968 | -0.39654 | 0.0072882 |
| metab_14976 | 0.98146 | -0.026999 | 0.0072882 |
| metab_2267 | 1.0795 | 0.11042 | 0.0072965 |
| metab_9193 | 0.81494 | -0.29523 | 0.0073374 |
| metab_10384 | 1.1163 | 0.15873 | 0.0073896 |
| metab_14051 | 0.95187 | -0.071159 | 0.0073975 |
| metab_11684 | 1.1532 | 0.20563 | 0.0074059 |
| Mandelic Acid | 1.0302 | 0.042952 | 0.0074497 |
| Threonylhydroxyproline | 2.3661 | 1.2425 | 0.0075471 |
| metab_8432 | 0.89559 | -0.15909 | 0.0075895 |
| metab_8829 | 1.1662 | 0.22177 | 0.0076478 |
| metab_537 | 1.4732 | 0.55891 | 0.0076551 |
| L-Pyroglutamyl-L-histidyl-3,3-dimethylprolinamide | 1.2913 | 0.36887 | 0.0076594 |
| metab_17553 | 0.95359 | -0.068559 | 0.0076901 |
| metab_2098 | 0.96541 | -0.050789 | 0.0077111 |
| Dehydrocrotonin | 1.2176 | 0.28399 | 0.0077257 |
| Fumitremorgin B | 1.3691 | 0.45322 | 0.0077621 |
| metab_6100 | 1.0192 | 0.027441 | 0.0077626 |
| metab_5125 | 1.521 | 0.60507 | 0.0077788 |
| metab_6529 | 1.0829 | 0.11494 | 0.0077884 |
| metab_1616 | 0.94918 | -0.075252 | 0.0078419 |
| metab_14274 | 0.86017 | -0.2173 | 0.0078629 |
| metab_9986 | 1.2954 | 0.37338 | 0.0079113 |
| N-(1-Methylheptyl)-N'-phenyl-1,4-benzenediamine | 1.0675 | 0.094291 | 0.0079179 |
| 6-methyl-5-Hepten-2-one | 1.1558 | 0.20893 | 0.0079318 |
| metab_14604 | 0.95523 | -0.066084 | 0.0079467 |
| metab_16589 | 0.95039 | -0.073408 | 0.0079813 |
| Deoxycholic acid 3-glucuronide | 1.0652 | 0.091131 | 0.0079959 |
| Mactraxanthin | 1.1337 | 0.18099 | 0.008013 |
| metab_10401 | 1.1633 | 0.21822 | 0.00802 |
| metab_13454 | 1.0803 | 0.11149 | 0.008048 |
| metab_7205 | 0.9383 | -0.091873 | 0.0080585 |
| metab_9375 | 1.1101 | 0.15073 | 0.0080679 |
| metab_7994 | 1.1817 | 0.24088 | 0.0080979 |
| metab_5318 | 1.1575 | 0.21099 | 0.0081009 |
| metab_13712 | 1.1713 | 0.22814 | 0.0081393 |
| metab_13671 | 3.8018 | 1.9267 | 0.0081727 |
| metab_10491 | 0.95489 | -0.066591 | 0.0081984 |
| A-Methapred | 1.0718 | 0.1 | 0.008212 |
| metab_2916 | 1.1392 | 0.18797 | 0.0082246 |
| metab_10474 | 1.0435 | 0.061385 | 0.0082289 |
| Paeonol | 1.0511 | 0.071958 | 0.0082326 |
| metab_14562 | 0.92611 | -0.11074 | 0.0083129 |
| metab_13453 | 1.0299 | 0.042494 | 0.0083299 |
| metab_3124 | 0.98399 | -0.023285 | 0.0083356 |
| metab_15540 | 1.1479 | 0.19896 | 0.0083524 |
| metab_8014 | 0.91481 | -0.12846 | 0.0083684 |
| PC(22:6(4Z,7Z,10Z,13Z,16Z,19Z)/16:0) | 0.99062 | -0.013593 | 0.0084083 |
| metab_16659 | 0.96325 | -0.054022 | 0.0084406 |
| metab_5335 | 1.1162 | 0.15861 | 0.0084557 |
| metab_10669 | 1.1002 | 0.13776 | 0.0084797 |
| metab_9094 | 2.2398 | 1.1634 | 0.0084907 |
| metab_14491 | 0.94181 | -0.086489 | 0.0085522 |
| metab_7266 | 0.7887 | -0.34246 | 0.0085829 |
| metab_8724 | 0.98697 | -0.018927 | 0.0085844 |
| metab_2910 | 1.0373 | 0.052813 | 0.0085862 |
| Kynurenic Acid | 1.0793 | 0.11007 | 0.0085972 |
| metab_7446 | 0.89479 | -0.16038 | 0.008644 |
| Diisobutyl phthalate | 1.1194 | 0.16275 | 0.0086547 |
| metab_5491 | 1.0544 | 0.076488 | 0.0086886 |
| metab_14186 | 2.0342 | 1.0245 | 0.0087302 |
| metab_11758 | 1.0506 | 0.071174 | 0.0087415 |
| metab_5091 | 0.96469 | -0.051858 | 0.0087819 |
| metab_1443 | 0.87992 | -0.18455 | 0.0087871 |
| metab_4283 | 1.1847 | 0.24446 | 0.0088187 |
| DG(10:0/8:0/0:0) | 0.84977 | -0.23485 | 0.0088776 |
| metab_10518 | 0.97832 | -0.031626 | 0.0088835 |
| Corchoroside A | 1.1059 | 0.14516 | 0.0088919 |
| Phenol sulphate | 1.0763 | 0.1061 | 0.008916 |
| metab_11481 | 1.2179 | 0.28435 | 0.0089191 |
| L-Aspartyl-L-Phenylalanine | 1.36 | 0.44363 | 0.008935 |
| metab_7185 | 1.0622 | 0.087025 | 0.0089581 |
| metab_3861 | 1.0453 | 0.063903 | 0.0090147 |
| Cortisone | 1.7429 | 0.80151 | 0.0090229 |
| 5-Hexyl-2-furanoctanoic acid | 0.85817 | -0.22066 | 0.0090287 |
| Methyl (3x,10R)-dihydroxy-11-dodecene-6,8-diynoate 10-glucoside | 1.5254 | 0.60917 | 0.0090314 |
| metab_8734 | 0.40796 | -1.2935 | 0.0090324 |
| metab_10631 | 0.96724 | -0.048052 | 0.0090395 |
| metab_15556 | 1.15 | 0.20162 | 0.009192 |
| metab_8571 | 1.7225 | 0.78451 | 0.0092161 |
| metab_13559 | 0.95603 | -0.064876 | 0.0092216 |
| metab_11795 | 1.0719 | 0.1002 | 0.0092249 |
| metab_4235 | 1.0563 | 0.078973 | 0.0092376 |
| metab_14217 | 0.60619 | -0.72217 | 0.0092892 |
| Sorbitan oleate | 1.0636 | 0.088944 | 0.0093306 |
| metab_13764 | 1.0218 | 0.03111 | 0.0093366 |
| metab_8034 | 1.1124 | 0.15371 | 0.009368 |
| metab_17074 | 1.0713 | 0.099317 | 0.0093857 |
| metab_14913 | 1.0323 | 0.045887 | 0.0094001 |
| metab_14066 | 0.89752 | -0.15598 | 0.0094115 |
| metab_10561 | 0.99022 | -0.014186 | 0.0094466 |
| metab_5050 | 0.97185 | -0.041192 | 0.009448 |
| metab_10671 | 0.97177 | -0.041314 | 0.0094743 |
| metab_15474 | 1.0409 | 0.05783 | 0.0094944 |
| metab_12441 | 0.98252 | -0.025439 | 0.0095188 |
| metab_3932 | 1.0666 | 0.092986 | 0.0095788 |
| L-trans-4-Methyl-2-pyrrolidinecarboxylic acid | 1.0454 | 0.064052 | 0.0095993 |
| Diguanosine diphosphate | 0.86811 | -0.20405 | 0.009613 |
| Tanabalin | 1.1361 | 0.18412 | 0.0097654 |
| metab_7570 | 0.97644 | -0.034391 | 0.0097871 |
| Lactulose | 1.0738 | 0.10267 | 0.0098039 |
| D-Apiose | 1.0147 | 0.021095 | 0.0098455 |
| metab_11806 | 1.0596 | 0.083584 | 0.0098656 |
| metab_9151 | 1.154 | 0.20667 | 0.0099343 |
| metab_9426 | 1.208 | 0.27267 | 0.0099365 |
| metab_3142 | 1.0806 | 0.11181 | 0.0099395 |
| metab_17709 | 0.88067 | -0.18332 | 0.0099501 |
| Diglyme | 1.2265 | 0.2946 | 0.0099671 |
| metab_13102 | 1.0481 | 0.067837 | 0.0099677 |
| metab_16861 | 1.1429 | 0.19269 | 0.0099902 |
| metab_9610 | 0.78552 | -0.34829 | 0.0099915 |
| L-Norleucine | 1.0086 | 0.012348 | 0.010049 |
| N,n-Dimethylguanosine | 1.0321 | 0.045513 | 0.010051 |
| metab_2685 | 1.0358 | 0.050734 | 0.010052 |
| metab_16699 | 1.9653 | 0.97477 | 0.01009 |
| DL-b-Hydroxycaprylic acid | 1.1441 | 0.19422 | 0.01012 |
| Gibberellin A43 | 1.0395 | 0.055951 | 0.010138 |
| metab_9194 | 0.89762 | -0.15582 | 0.010148 |
| metab_14671 | 0.98884 | -0.016188 | 0.010171 |
| metab_17567 | 0.91466 | -0.1287 | 0.010171 |
| metab_16402 | 1.1363 | 0.1844 | 0.010184 |
| metab_6330 | 0.83358 | -0.26261 | 0.01019 |
| metab_503 | 1.1339 | 0.18125 | 0.010224 |
| metab_7198 | 1.6872 | 0.75463 | 0.010235 |
| 2-Deoxy-D-Ribose | 1.0196 | 0.028073 | 0.010255 |
| metab_5654 | 0.96057 | -0.058037 | 0.010324 |
| metab_8601 | 0.97884 | -0.030858 | 0.010345 |
| DG(8:0/10:0/0:0) | 0.85993 | -0.2177 | 0.010382 |
| metab_15286 | 1.0483 | 0.067989 | 0.01044 |
| metab_2819 | 0.95558 | -0.065549 | 0.010547 |
| metab_14523 | 0.93839 | -0.091739 | 0.010567 |
| Tyrosyl-Serine | 1.1006 | 0.1383 | 0.010631 |
| metab_8759 | 0.9022 | -0.14848 | 0.010685 |
| metab_14148 | 0.95009 | -0.073859 | 0.010703 |
| Thyroxine | 0.97572 | -0.035454 | 0.010707 |
| metab_3615 | 0.89521 | -0.1597 | 0.010708 |
| metab_10554 | 0.9536 | -0.068547 | 0.010708 |
| metab_1278 | 1.0731 | 0.10174 | 0.010727 |
| metab_2786 | 0.66291 | -0.59312 | 0.010735 |
| Cis-Vaccenic acid | 1.242 | 0.31272 | 0.010746 |
| Ogyline | 1.1832 | 0.24267 | 0.010749 |
| metab_9209 | 1.3409 | 0.42318 | 0.010782 |
| metab_6931 | 0.95926 | -0.060008 | 0.010803 |
| metab_12202 | 1.2484 | 0.32009 | 0.010884 |
| TG(10:0/8:0/8:0) | 0.85462 | -0.22665 | 0.010885 |
| metab_17310 | 0.97905 | -0.030549 | 0.010956 |
| metab_1138 | 1.0922 | 0.12724 | 0.010998 |
| metab_16671 | 1.2563 | 0.32916 | 0.011009 |
| metab_14448 | 0.98261 | -0.02531 | 0.011024 |
| metab_8406 | 0.89115 | -0.16626 | 0.011026 |
| metab_4833 | 2.8232 | 1.4973 | 0.011088 |
| metab_8224 | 1.1618 | 0.21638 | 0.01111 |
| Phthalide | 1.3486 | 0.43149 | 0.011128 |
| metab_13901 | 0.94416 | -0.082902 | 0.011147 |
| metab_7460 | 0.97372 | -0.038427 | 0.011329 |
| metab_13796 | 0.94497 | -0.081656 | 0.011401 |
| Donhexocin | 1.1146 | 0.15656 | 0.011484 |
| DG(2:0/0:0/20:4(6E,8Z,11Z,13E)-2OH(5S,15S)) | 1.0616 | 0.086286 | 0.011485 |
| metab_9680 | 1.1223 | 0.16651 | 0.011543 |
| 9,10-Epoxy-18-hydroxy-octadecanoic acid | 1.043 | 0.060753 | 0.011556 |
| metab_11353 | 0.94563 | -0.080656 | 0.011561 |
| metab_3244 | 0.82115 | -0.28429 | 0.011571 |
| metab_7441 | 0.9731 | -0.039346 | 0.011586 |
| metab_12913 | 0.94097 | -0.087785 | 0.011649 |
| metab_8392 | 1.5909 | 0.66981 | 0.011655 |
| metab_13588 | 0.94955 | -0.074682 | 0.011735 |
| metab_9613 | 0.97036 | -0.043401 | 0.011741 |
| metab_10339 | 0.88896 | -0.16981 | 0.011787 |
| metab_7442 | 0.95458 | -0.067066 | 0.011818 |
| metab_12912 | 0.87774 | -0.18814 | 0.011818 |
| Cis-Caffeic acid | 1.1365 | 0.18465 | 0.011893 |
| metab_10014 | 0.86052 | -0.21672 | 0.01191 |
| metab_12965 | 1.0417 | 0.058933 | 0.011933 |
| metab_12253 | 1.0896 | 0.12376 | 0.011965 |
| metab_10283 | 1.2604 | 0.33392 | 0.011971 |
| metab_14785 | 0.94341 | -0.08405 | 0.011972 |
| metab_8245 | 1.1031 | 0.14159 | 0.012015 |
| Fluorouracil | 0.83505 | -0.26006 | 0.01202 |
| metab_2901 | 1.2976 | 0.37587 | 0.01208 |
| metab_13619 | 0.91381 | -0.13004 | 0.012085 |
| metab_4202 | 1.0165 | 0.023554 | 0.012088 |
| Histamine-betaxanthin | 0.95421 | -0.067624 | 0.012107 |
| Fructosyl valine | 1.0943 | 0.13001 | 0.012115 |
| metab_8254 | 1.583 | 0.6627 | 0.012116 |
| Dioctyl Phthalate | 1.0813 | 0.11275 | 0.012121 |
| metab_1683 | 1.0586 | 0.082193 | 0.012136 |
| metab_7726 | 1.0338 | 0.047989 | 0.012157 |
| metab_12303 | 1.1545 | 0.20726 | 0.012242 |
| metab_11376 | 0.90713 | -0.14061 | 0.012281 |
| metab_8509 | 0.90249 | -0.14802 | 0.012388 |
| metab_2016 | 1.1212 | 0.16498 | 0.012399 |
| Lopinavir | 1.13 | 0.17636 | 0.012495 |
| metab_13761 | 0.96321 | -0.054073 | 0.012503 |
| metab_16462 | 0.98248 | -0.025502 | 0.012553 |
| metab_10585 | 0.9673 | -0.047958 | 0.012562 |
| metab_9730 | 1.2515 | 0.3237 | 0.012636 |
| metab_8779 | 1.4219 | 0.50785 | 0.012642 |
| metab_1320 | 1.0447 | 0.06309 | 0.012671 |
| metab_1473 | 0.94748 | -0.077835 | 0.012768 |
| metab_9433 | 1.0371 | 0.052624 | 0.012777 |
| metab_13830 | 0.94808 | -0.076914 | 0.012805 |
| Arenobufagin | 1.2435 | 0.31436 | 0.012812 |
| 3a,21-Dihydroxy-5b-pregnane-11,20-dione | 1.0437 | 0.061706 | 0.012813 |
| metab_16205 | 0.94411 | -0.082974 | 0.012815 |
| 1,5-Naphthalenediamine | 1.0878 | 0.12145 | 0.012831 |
| metab_15862 | 1.1357 | 0.18364 | 0.012858 |
| metab_1523 | 0.9804 | -0.02856 | 0.01289 |
| metab_8396 | 0.83281 | -0.26394 | 0.01293 |
| DG(8:0/8:0/0:0) | 0.84904 | -0.2361 | 0.012934 |
| metab_14582 | 0.95469 | -0.066894 | 0.012958 |
| (17E,19E,21E,23E,25E)-4,6,8,10,12,14,16,27-Octahydroxy-3-(1-hydroxyhexyl)-17,28-dimethyl-1-oxacyclooctacosa-17,19,21,23,25-pentaen-2-one | 1.1069 | 0.14652 | 0.012998 |
| 19-Hydroxycinnzeylanol 19-glucoside | 1.0842 | 0.11661 | 0.013017 |
| metab_10640 | 0.97092 | -0.04257 | 0.013037 |
| metab_15881 | 1.1657 | 0.2212 | 0.013066 |
| metab_8506 | 1.0391 | 0.055275 | 0.013121 |
| metab_14401 | 1.1238 | 0.16843 | 0.013139 |
| metab_5070 | 0.97712 | -0.033386 | 0.013187 |
| metab_4326 | 1.4265 | 0.51248 | 0.013232 |
| metab_2001 | 1.0647 | 0.090395 | 0.013274 |
| metab_13166 | 1.0939 | 0.12949 | 0.013324 |
| metab_9526 | 1.1328 | 0.1799 | 0.013384 |
| metab_627 | 1.3059 | 0.38507 | 0.013413 |
| metab_10835 | 0.92738 | -0.10877 | 0.01349 |
| metab_3901 | 1.1111 | 0.15199 | 0.013534 |
| metab_12223 | 1.0911 | 0.12583 | 0.01356 |
| metab_1135 | 1.1378 | 0.1862 | 0.013566 |
| metab_16660 | 0.96473 | -0.051809 | 0.013639 |
| metab_11590 | 1.0216 | 0.030774 | 0.013648 |
| metab_2263 | 1.0841 | 0.11653 | 0.013669 |
| metab_13572 | 1.2678 | 0.34238 | 0.01369 |
| metab_1039 | 0.92321 | -0.11527 | 0.013701 |
| metab_16761 | 1.1916 | 0.25295 | 0.013716 |
| metab_15047 | 1.0451 | 0.063591 | 0.013755 |
| SM(d18:2(4E,14Z)/14:0) | 0.9758 | -0.035337 | 0.013801 |
| Fructosylvaline | 1.0891 | 0.12313 | 0.013809 |
| metab_1230 | 0.9469 | -0.078714 | 0.013918 |
| metab_3900 | 1.2085 | 0.27319 | 0.013962 |
| metab_2999 | 1.1063 | 0.1458 | 0.013973 |
| metab_14578 | 0.94205 | -0.086119 | 0.014018 |
| metab_11534 | 1.0909 | 0.12558 | 0.014034 |
| metab_15036 | 0.9654 | -0.050799 | 0.014037 |
| metab_1391 | 0.97602 | -0.035011 | 0.014062 |
| metab_16324 | 1.3331 | 0.41477 | 0.014064 |
| metab_17389 | 1.0704 | 0.098132 | 0.014093 |
| metab_4735 | 0.91461 | -0.12877 | 0.014179 |
| metab_8000 | 0.95013 | -0.07381 | 0.014286 |
| metab_13557 | 1.3236 | 0.40447 | 0.014335 |
| metab_9819 | 1.3089 | 0.3884 | 0.014347 |
| metab_14536 | 1.2785 | 0.35446 | 0.014402 |
| N-Acetyl-D-tryptophan | 1.1033 | 0.14185 | 0.014439 |
| metab_1055 | 1.0225 | 0.032107 | 0.014537 |
| metab_7999 | 0.94398 | -0.083172 | 0.014562 |
| Debenzoylzucchini factor B | 1.0947 | 0.13049 | 0.014565 |
| metab_3060 | 0.85805 | -0.22086 | 0.014568 |
| Artemorin | 1.0197 | 0.028095 | 0.014573 |
| metab_13751 | 0.95655 | -0.064093 | 0.014592 |
| metab_1162 | 1.1275 | 0.17308 | 0.014596 |
| metab_7431 | 0.9772 | -0.033279 | 0.01462 |
| (S)-5-Amino-3-oxohexanoate | 1.0134 | 0.019215 | 0.014627 |
| metab_10241 | 1.1304 | 0.17679 | 0.014667 |
| (3-{[3-(2-Amino-2-Oxoethyl)-1-Benzyl-2-Ethyl-1h-Indol-5-Yl]oxy}propyl)phosphonic Acid | 1.3263 | 0.40741 | 0.014681 |
| metab_6939 | 0.97998 | -0.029171 | 0.014699 |
| metab_966 | 1.0438 | 0.061816 | 0.014749 |
| metab_5560 | 1.0546 | 0.076681 | 0.014761 |
| metab_3784 | 0.932 | -0.10161 | 0.014765 |
| (10S,11S)-Pterosin C | 1.1651 | 0.22041 | 0.014802 |
| 9-(3-Methyl-5-propylfuran-2-yl)nonanoylcarnitine | 1.0568 | 0.079729 | 0.014808 |
| metab_13820 | 0.98602 | -0.020304 | 0.014831 |
| metab_17200 | 1.6499 | 0.72241 | 0.014831 |
| 2-Hydroxypropyl octanoate | 1.0104 | 0.014946 | 0.014845 |
| metab_15729 | 0.98335 | -0.024227 | 0.014845 |
| Gamma-glutamyl-L-putrescine | 1.0556 | 0.07811 | 0.014949 |
| metab_13941 | 0.74866 | -0.41762 | 0.014952 |
| metab_4198 | 0.99343 | -0.0095036 | 0.01496 |
| PI(16:1(9Z)/20:4(5Z,7E,11Z,14Z)-OH(9)) | 0.93013 | -0.1045 | 0.014977 |
| metab_9795 | 1.1857 | 0.24574 | 0.01499 |
| Methylprednisolone succinate | 1.2848 | 0.36151 | 0.015016 |
| metab_12836 | 0.96048 | -0.058176 | 0.015039 |
| metab_13514 | 1.1037 | 0.14233 | 0.015052 |
| Leu Ile | 0.87761 | -0.18835 | 0.015067 |
| metab_9222 | 0.98798 | -0.017445 | 0.0151 |
| metab_8307 | 0.93544 | -0.09628 | 0.015104 |
| 2-Polyprenyl-3-methyl-5-hydroxy-6-methoxy-1,4-benzoquinone | 1.2416 | 0.31216 | 0.015115 |
| TG(8:0/8:0/8:0) | 0.84239 | -0.24744 | 0.01516 |
| metab_7526 | 1.1126 | 0.15388 | 0.015175 |
| (5R,6S)-3-(2-Carbamoyloxyethylsulfanyl)-6-(1-hydroxyethyl)-7-oxo-4-thia-1-azabicyclo[3.2.0]hept-2-ene-2-carboxylic acid | 1.2378 | 0.30782 | 0.015185 |
| metab_14058 | 0.90238 | -0.1482 | 0.015279 |
| Serylalanine | 1.0568 | 0.079638 | 0.015292 |
| metab_1549 | 1.3644 | 0.44824 | 0.015346 |
| metab_13470 | 1.0701 | 0.097745 | 0.015347 |
| metab_16949 | 1.5056 | 0.59034 | 0.015361 |
| metab_15213 | 1.0347 | 0.049147 | 0.015391 |
| metab_16801 | 1.049 | 0.069029 | 0.015522 |
| 11b-Hydroxyprogesterone | 1.0439 | 0.062033 | 0.015532 |
| metab_17619 | 0.97978 | -0.029472 | 0.015536 |
| metab_13775 | 1.408 | 0.49365 | 0.015546 |
| metab_14734 | 1.1633 | 0.21819 | 0.015568 |
| Alpha-CEHC glucuronide | 1.0903 | 0.12469 | 0.015636 |
| metab_8291 | 0.94648 | -0.079361 | 0.0157 |
| metab_9953 | 0.94794 | -0.077138 | 0.015728 |
| metab_8246 | 0.95109 | -0.072349 | 0.015734 |
| metab_8410 | 0.62787 | -0.67146 | 0.015743 |
| metab_14496 | 0.89517 | -0.15976 | 0.015772 |
| metab_13531 | 0.9618 | -0.056195 | 0.015807 |
| 1,2-Dihydronaphthalene-1,2-diol | 0.98584 | -0.020579 | 0.015915 |
| 11beta,20-Dihydroxy-3-oxopregn-4-en-21-oic acid | 1.1858 | 0.24583 | 0.015992 |
| metab_12090 | 1.074 | 0.10301 | 0.016008 |
| metab_17002 | 1.1741 | 0.2315 | 0.016066 |
| metab_9743 | 1.0578 | 0.081071 | 0.016127 |
| Methyl 20-dihydroprednisolonate | 1.189 | 0.24973 | 0.016164 |
| metab_8223 | 1.4177 | 0.50352 | 0.016206 |
| metab_11296 | 0.96688 | -0.048588 | 0.016228 |
| metab_1268 | 0.96452 | -0.052119 | 0.016251 |
| metab_1451 | 0.95787 | -0.062103 | 0.016324 |
| 9,12,13-TriHOME | 1.018 | 0.025753 | 0.016344 |
| metab_13306 | 0.86125 | -0.21549 | 0.01637 |
| metab_194 | 1.1207 | 0.16444 | 0.016371 |
| metab_2196 | 0.93683 | -0.094137 | 0.016407 |
| metab_12095 | 1.1882 | 0.24877 | 0.016548 |
| metab_6976 | 1.1141 | 0.15588 | 0.016659 |
| metab_13120 | 1.1164 | 0.15883 | 0.016666 |
| metab_8876 | 0.66626 | -0.58585 | 0.016726 |
| metab_4490 | 1.1448 | 0.19512 | 0.01685 |
| metab_3055 | 1.0511 | 0.071938 | 0.016854 |
| Kahweol | 1.3934 | 0.47862 | 0.016882 |
| metab_14663 | 1.2852 | 0.36196 | 0.016945 |
| 2-Acetolactate | 1.0254 | 0.036212 | 0.017011 |
| 5-Methoxyindoleacetate | 1.1388 | 0.18746 | 0.017038 |
| LysoPC(0:0/16:0) | 0.97326 | -0.039103 | 0.017174 |
| Cer(d20:1/18:3(9,11,15)-OH(13)) | 1.2083 | 0.27296 | 0.017227 |
| metab_14106 | 1.4484 | 0.5345 | 0.017261 |
| Isoleucylproline | 1.0716 | 0.099748 | 0.017278 |
| L-Monomenthyl glutarate | 1.0315 | 0.044766 | 0.017328 |
| Dihydrocortisol | 0.96665 | -0.048938 | 0.017363 |
| metab_7973 | 0.97341 | -0.03888 | 0.017377 |
| Ingenol mebutate | 1.2913 | 0.36883 | 0.017469 |
| metab_5700 | 1.0407 | 0.05758 | 0.017473 |
| Phenylacetylglutamine | 1.0423 | 0.059706 | 0.017482 |
| metab_8568 | 0.83577 | -0.25882 | 0.017534 |
| metab_2714 | 1.1295 | 0.17567 | 0.017534 |
| metab_10613 | 0.9789 | -0.030766 | 0.017618 |
| metab_8983 | 1.4233 | 0.50928 | 0.017664 |
| (-)-Arctigenin | 1.1562 | 0.20938 | 0.017675 |
| Macrophorin B | 1.1516 | 0.20365 | 0.017677 |
| metab_1118 | 1.1361 | 0.18411 | 0.017827 |
| metab_8252 | 0.74534 | -0.42403 | 0.017833 |
| Linoleoyl ethanolamide | 1.0244 | 0.034807 | 0.017833 |
| metab_2 | 1.023 | 0.032736 | 0.017869 |
| metab_1267 | 0.91984 | -0.12054 | 0.017923 |
| metab_10477 | 0.92382 | -0.11431 | 0.017979 |
| 5-Benzylacyclouridine | 1.2205 | 0.28744 | 0.018041 |
| metab_9523 | 0.91902 | -0.12183 | 0.018159 |
| metab_1058 | 1.0224 | 0.03202 | 0.018201 |
| metab_2854 | 1.0292 | 0.041519 | 0.018203 |
| metab_16636 | 0.96074 | -0.05778 | 0.018302 |
| DG(8:0/PGE2/0:0) | 1.1159 | 0.15816 | 0.018338 |
| metab_8017 | 0.9592 | -0.060098 | 0.018352 |
| metab_5037 | 0.83379 | -0.26224 | 0.018392 |
| metab_9605 | 1.5988 | 0.67696 | 0.018443 |
| metab_9102 | 1.3193 | 0.39978 | 0.018452 |
| Grifolic acid | 1.0683 | 0.095317 | 0.018454 |
| metab_12957 | 0.85577 | -0.2247 | 0.018473 |
| metab_8314 | 0.97394 | -0.038096 | 0.018487 |
| metab_15492 | 1.0728 | 0.10142 | 0.018503 |
| Anthranilic acid | 1.0365 | 0.051767 | 0.018597 |
| metab_4194 | 1.042 | 0.059381 | 0.018651 |
| 9-OxoODE | 1.3288 | 0.41015 | 0.018666 |
| metab_3778 | 1.0812 | 0.11264 | 0.018671 |
| metab_3776 | 0.9522 | -0.070667 | 0.018722 |
| metab_6361 | 1.01 | 0.014374 | 0.01875 |
| N'-Formylkynurenine | 0.92653 | -0.1101 | 0.018774 |
| metab_8181 | 0.88153 | -0.18192 | 0.018792 |
| Leucylproline | 1.0352 | 0.04993 | 0.018858 |
| metab_3236 | 1.0385 | 0.054485 | 0.018877 |
| metab_1057 | 1.0233 | 0.033164 | 0.018903 |
| 6-[2,3-Dihydroxy-1-(hydroxymethyl)propyl]-1,2-dihydro-7-hydroxy-9-methoxy-cyclopenta[c][1]benzopyran-3,4-dione | 1.0684 | 0.095487 | 0.018932 |
| metab_14355 | 0.87837 | -0.1871 | 0.018965 |
| metab_8270 | 0.96449 | -0.052161 | 0.019001 |
| metab_9229 | 1.398 | 0.48337 | 0.019003 |
| Phenylalanylaspartic acid | 1.1651 | 0.22041 | 0.019006 |
| metab_552 | 1.0586 | 0.082182 | 0.019006 |
| 5,5'-Dithiobis(2-nitrobenzoic acid) | 1.2179 | 0.28437 | 0.019102 |
| 3-Isopropylmalic acid | 0.9655 | -0.050659 | 0.019111 |
| SM(d18:2(4E,14Z)/16:0) | 0.98917 | -0.015703 | 0.019207 |
| Lactose | 1.0578 | 0.081041 | 0.019228 |
| metab_10460 | 0.89037 | -0.16752 | 0.019292 |
| (-)-Sparteine | 1.073 | 0.10167 | 0.019326 |
| Galactocerebroside | 0.97468 | -0.036998 | 0.019404 |
| N-Methyl-2-[3-(trifluoromethyl)phenyl]-3,4-dihydropyrazol-5-amine | 0.94334 | -0.084154 | 0.019411 |
| metab_7753 | 0.96257 | -0.055039 | 0.019421 |
| metab_3047 | 0.80328 | -0.31603 | 0.019433 |
| metab_14603 | 1.0773 | 0.10749 | 0.019439 |
| Lycorine | 1.2465 | 0.31792 | 0.019512 |
| Chalcone | 1.0338 | 0.047993 | 0.019527 |
| Prostratin | 1.2169 | 0.28325 | 0.019558 |
| metab_5629 | 1.0712 | 0.099166 | 0.019573 |
| 3-(1-Pyrazolyl)-Alanine | 1.4229 | 0.50888 | 0.019583 |
| metab_12205 | 2.0351 | 1.0251 | 0.019584 |
| Tyrosol 4-sulfate | 1.5614 | 0.64284 | 0.019613 |
| 3-(Aminomethyl)-2,5,9-trimethyl-7H-furo[3,2-g]chromen-7-one | 1.026 | 0.037067 | 0.019617 |
| (-)-2-Difluoromethylornithine | 1.1418 | 0.19127 | 0.019738 |
| metab_14850 | 0.92987 | -0.10489 | 0.019766 |
| metab_388 | 1.5005 | 0.58545 | 0.019799 |
| metab_5346 | 1.0358 | 0.050748 | 0.019806 |
| Metazin | 0.84431 | -0.24415 | 0.019896 |
| metab_2063 | 1.0986 | 0.13569 | 0.020018 |
| metab_8757 | 0.69512 | -0.52466 | 0.020036 |
| metab_9189 | 0.86459 | -0.20992 | 0.020044 |
| metab_5136 | 1.0706 | 0.098391 | 0.020048 |
| metab_8697 | 1.1599 | 0.214 | 0.020072 |
| metab_17123 | 1.0388 | 0.054948 | 0.020091 |
| metab_2756 | 1.0874 | 0.12082 | 0.020111 |
| metab_8211 | 0.92768 | -0.1083 | 0.020284 |
| metab_16558 | 0.93398 | -0.09853 | 0.020383 |
| metab_16071 | 1.2104 | 0.27552 | 0.020387 |
| metab_16162 | 1.4834 | 0.56892 | 0.020431 |
| metab_13696 | 0.9812 | -0.02738 | 0.020476 |
| metab_1213 | 1.0568 | 0.079702 | 0.020528 |
| metab_12829 | 0.94306 | -0.084573 | 0.02056 |
| metab_14552 | 1.0709 | 0.098778 | 0.020573 |
| metab_11412 | 1.3021 | 0.38086 | 0.020597 |
| metab_10625 | 0.86433 | -0.21034 | 0.020605 |
| metab_10085 | 1.0433 | 0.061202 | 0.020621 |
| metab_8750 | 0.96899 | -0.04544 | 0.020647 |
| metab_4441 | 1.0904 | 0.12485 | 0.020697 |
| 3-Hydroxydodeca-7,10-dienoylcarnitine | 1.0414 | 0.058543 | 0.020755 |
| metab_3351 | 1.0398 | 0.056373 | 0.020764 |
| metab_8487 | 0.92171 | -0.11762 | 0.020807 |
| 11,13-Dihydrotaraxinic acid glucosyl ester | 1.0564 | 0.079196 | 0.020832 |
| metab_4869 | 0.92389 | -0.11421 | 0.020867 |
| Leu Asp | 1.042 | 0.059424 | 0.020906 |
| metab_1928 | 1.1166 | 0.15905 | 0.020941 |
| metab_14970 | 0.93347 | -0.099318 | 0.021007 |
| metab_13631 | 0.93156 | -0.10229 | 0.021012 |
| metab_7327 | 0.97317 | -0.039241 | 0.021013 |
| metab_10831 | 0.96291 | -0.054532 | 0.021045 |
| metab_1104 | 1.0364 | 0.051607 | 0.021054 |
| metab_11892 | 1.0746 | 0.10384 | 0.021073 |
| metab_11432 | 1.0504 | 0.070927 | 0.02115 |
| Propafenone | 2.2206 | 1.1509 | 0.021233 |
| metab_3835 | 1.0288 | 0.040911 | 0.021237 |
| metab_7725 | 1.118 | 0.16094 | 0.021239 |
| metab_7452 | 0.97804 | -0.032037 | 0.021304 |
| metab_2061 | 1.034 | 0.048305 | 0.02135 |
| metab_15531 | 0.92732 | -0.10887 | 0.021395 |
| Obacunone | 1.1809 | 0.23993 | 0.021401 |
| metab_9695 | 0.95484 | -0.066667 | 0.021543 |
| metab_3082 | 1.0261 | 0.037146 | 0.021582 |
| metab_11269 | 0.97486 | -0.036733 | 0.021584 |
| metab_8703 | 1.6459 | 0.71888 | 0.021642 |
| metab_3159 | 0.96186 | -0.056097 | 0.021693 |
| 9-Hydroxy-10-O-D-glucuronoside-12Z-octadecenoate | 1.067 | 0.093616 | 0.021716 |
| metab_17454 | 0.95253 | -0.070163 | 0.021734 |
| metab_13685 | 2.4449 | 1.2898 | 0.021741 |
| metab_2772 | 0.87613 | -0.19078 | 0.021771 |
| metab_10075 | 1.0216 | 0.030881 | 0.021815 |
| metab_14063 | 1.2617 | 0.33538 | 0.02184 |
| metab_6494 | 1.0705 | 0.098318 | 0.021863 |
| metab_9587 | 0.94122 | -0.087394 | 0.021901 |
| metab_14685 | 1.1297 | 0.17594 | 0.021983 |
| metab_2764 | 1.4045 | 0.49007 | 0.021999 |
| metab_16504 | 0.97577 | -0.03538 | 0.02202 |
| metab_9827 | 0.89116 | -0.16624 | 0.022045 |
| metab_14184 | 0.95998 | -0.058924 | 0.02205 |
| metab_2223 | 1.0439 | 0.061923 | 0.022125 |
| metab_17506 | 1.4141 | 0.49987 | 0.022156 |
| 5-(2'-Carboxyethyl)-4,6-Dihydroxypicolinate | 1.0296 | 0.04214 | 0.022207 |
| Dibutyl Phthalate | 1.0905 | 0.12496 | 0.022313 |
| Allysine | 1.0184 | 0.026249 | 0.022383 |
| LysoPC(0:0/18:0) | 0.95635 | -0.064389 | 0.02239 |
| metab_12135 | 1.0857 | 0.11865 | 0.022418 |
| metab_7535 | 0.68293 | -0.5502 | 0.022467 |
| metab_6412 | 1.0327 | 0.046383 | 0.022531 |
| metab_11732 | 1.1607 | 0.21498 | 0.022564 |
| metab_16676 | 0.92579 | -0.11125 | 0.022798 |
| PS(15:0/22:2(13Z,16Z)) | 0.84173 | -0.24857 | 0.022897 |
| metab_3346 | 1.0608 | 0.085185 | 0.022948 |
| metab_8819 | 0.91705 | -0.12493 | 0.022953 |
| metab_1491 | 1.4446 | 0.53069 | 0.022976 |
| metab_11784 | 1.1052 | 0.14436 | 0.022999 |
| metab_14150 | 0.72129 | -0.47135 | 0.023005 |
| Glucose lactate acetate | 0.94002 | -0.089232 | 0.023022 |
| metab_3219 | 1.0275 | 0.039105 | 0.023079 |
| metab_12847 | 0.95107 | -0.072381 | 0.023093 |
| 8H-Purin-8-one, 6-amino-7,9-dihydro-2-((1S)-1-methylbutoxy)-9-(5-(1-piperidinyl)pentyl)- | 1.0613 | 0.085815 | 0.023256 |
| metab_12066 | 1.037 | 0.05236 | 0.023292 |
| Dmean | 1.049 | 0.06904 | 0.023317 |
| metab_14776 | 1.1587 | 0.21254 | 0.023392 |
| metab_3151 | 0.94526 | -0.081214 | 0.023422 |
| metab_7093 | 1.0869 | 0.12016 | 0.023486 |
| metab_10596 | 0.97486 | -0.03673 | 0.023551 |
| (+)-cis-5,6-Dihydro-5-hydroxy-4-methoxy-6-(2-phenylethyl)-2H-pyran-2-one | 2.1647 | 1.1142 | 0.023553 |
| metab_5057 | 0.97727 | -0.033166 | 0.023585 |
| metab_8677 | 0.95879 | -0.06071 | 0.023609 |
| metab_9446 | 1.6684 | 0.73845 | 0.023616 |
| metab_9014 | 1.1347 | 0.18233 | 0.023635 |
| metab_15183 | 1.0874 | 0.12087 | 0.02366 |
| metab_13590 | 0.9861 | -0.020189 | 0.023748 |
| metab_2908 | 1.092 | 0.12694 | 0.023915 |
| metab_9600 | 0.94956 | -0.074674 | 0.023991 |
| metab_11406 | 0.93141 | -0.10251 | 0.024012 |
| Sufentanil | 0.84567 | -0.24184 | 0.024015 |
| metab_13542 | 0.95192 | -0.071093 | 0.024057 |
| metab_9044 | 0.79367 | -0.33338 | 0.024159 |
| Algestone | 1.1731 | 0.23037 | 0.024201 |
| metab_13621 | 1.1404 | 0.18956 | 0.024238 |
| metab_14163 | 1.3755 | 0.45999 | 0.024279 |
| metab_16973 | 1.1164 | 0.1589 | 0.024287 |
| metab_8445 | 1.1892 | 0.24994 | 0.024412 |
| metab_8294 | 0.9561 | -0.06477 | 0.02446 |
| metab_1432 | 0.82457 | -0.27829 | 0.024548 |
| metab_5228 | 1.0489 | 0.068828 | 0.024571 |
| metab_12272 | 1.0681 | 0.094994 | 0.024607 |
| metab_13615 | 0.94711 | -0.078393 | 0.024612 |
| metab_14059 | 1.0472 | 0.066475 | 0.024662 |
| metab_13711 | 1.1541 | 0.20676 | 0.024677 |
| 2-(4-methylphenyl)-N-(4-propan-2-ylphenyl)imidazo[1,2-a]pyrazin-3-amine | 1.2694 | 0.34414 | 0.02468 |
| metab_11655 | 1.1357 | 0.1836 | 0.024787 |
| metab_10862 | 0.95569 | -0.065386 | 0.02482 |
| metab_13262 | 1.0382 | 0.054116 | 0.024826 |
| metab_15152 | 1.0235 | 0.033476 | 0.024901 |
| metab_53 | 0.829 | -0.27055 | 0.024951 |
| metab_3040 | 1.2157 | 0.28178 | 0.024981 |
| 5-Hydroxydec-8-enoylcarnitine | 1.0344 | 0.048759 | 0.025021 |
| metab_8925 | 0.96579 | -0.050225 | 0.025059 |
| metab_15167 | 1.2311 | 0.29998 | 0.02506 |
| 17-methylnonadecanoylcarnitine | 0.97606 | -0.034954 | 0.025133 |
| metab_14029 | 1.1193 | 0.16264 | 0.025249 |
| metab_586 | 1.0987 | 0.13579 | 0.025269 |
| metab_3469 | 1.0705 | 0.098323 | 0.025332 |
| metab_6532 | 1.0494 | 0.069611 | 0.025342 |
| metab_1905 | 1.0307 | 0.043647 | 0.025362 |
| metab_12313 | 1.0752 | 0.10465 | 0.025455 |
| metab_5127 | 0.95556 | -0.065577 | 0.02547 |
| metab_5329 | 0.97577 | -0.035382 | 0.025494 |
| metab_1700 | 0.98072 | -0.028089 | 0.025554 |
| metab_10129 | 1.2427 | 0.31342 | 0.025605 |
| PE(20:3(5Z,8Z,11Z)/20:5(5Z,8Z,11Z,14Z,17Z)) | 0.95095 | -0.072551 | 0.025655 |
| metab_1352 | 0.94581 | -0.080377 | 0.025662 |
| Xi-7-Hydroxyhexadecanedioic acid | 0.91922 | -0.12152 | 0.025723 |
| metab_17592 | 0.94601 | -0.080074 | 0.025743 |
| (2E)-Decenoyl-ACP | 1.1366 | 0.18468 | 0.025791 |
| metab_4316 | 0.958 | -0.061903 | 0.025798 |
| PS(22:6(4Z,7Z,10Z,13Z,16Z,19Z)/18:0) | 1.1087 | 0.14886 | 0.025844 |
| metab_10527 | 1.0843 | 0.11676 | 0.025873 |
| metab_15229 | 1.1591 | 0.21299 | 0.025894 |
| metab_11133 | 0.98819 | -0.017139 | 0.025918 |
| metab_7783 | 1.0754 | 0.10492 | 0.026026 |
| metab_14967 | 0.95883 | -0.060653 | 0.02604 |
| 2,3,4,5-Tetrahydro-2-pyridinecarboxylic acid | 1.0611 | 0.085593 | 0.026063 |
| metab_17327 | 1.0121 | 0.017321 | 0.026183 |
| metab_6921 | 1.2295 | 0.29803 | 0.026185 |
| metab_9715 | 0.98399 | -0.023287 | 0.026253 |
| PE(15:0/18:2(9Z,12Z)) | 1.0503 | 0.070787 | 0.026281 |
| metab_11893 | 1.036 | 0.051077 | 0.026282 |
| DIMP | 1.2076 | 0.27217 | 0.026351 |
| Xylobiose | 1.2576 | 0.33068 | 0.026458 |
| PE(P-18:0/0:0) | 0.97415 | -0.037788 | 0.026509 |
| metab_14499 | 0.9862 | -0.020055 | 0.02659 |
| metab_9817 | 1.1621 | 0.21669 | 0.026617 |
| metab_16455 | 1.1659 | 0.22147 | 0.026757 |
| Pantothenic Acid | 1.0295 | 0.041945 | 0.02678 |
| metab_292 | 1.0533 | 0.074866 | 0.026791 |
| metab_11752 | 1.0361 | 0.051155 | 0.026818 |
| metab_11383 | 0.91992 | -0.12042 | 0.026871 |
| Thiodi-glycolic acid | 0.93292 | -0.10018 | 0.026879 |
| metab_2920 | 1.0899 | 0.12416 | 0.026892 |
| metab_14972 | 1.0521 | 0.07328 | 0.026892 |
| metab_9454 | 2.0315 | 1.0225 | 0.026908 |
| metab_8944 | 1.2738 | 0.34912 | 0.026943 |
| metab_9634 | 0.81782 | -0.29014 | 0.026951 |
| Glutamyltyrosine | 1.0591 | 0.082834 | 0.026979 |
| All-trans-18-Hydroxyretinoic acid | 1.1644 | 0.21955 | 0.027004 |
| 4-Acetamidobutanoate | 1.0253 | 0.036073 | 0.027008 |
| metab_15008 | 0.9366 | -0.094494 | 0.027035 |
| metab_14408 | 0.98496 | -0.021858 | 0.027038 |
| metab_5263 | 1.0335 | 0.047483 | 0.027061 |
| 3-Nitrotyrosine | 0.95762 | -0.06248 | 0.027071 |
| metab_14577 | 0.98741 | -0.018283 | 0.027099 |
| metab_9494 | 1.1376 | 0.18596 | 0.027103 |
| metab_7549 | 0.9678 | -0.047218 | 0.027235 |
| metab_15566 | 0.94009 | -0.089126 | 0.027239 |
| metab_1339 | 1.0819 | 0.11358 | 0.02725 |
| metab_3270 | 0.97244 | -0.040325 | 0.027277 |
| metab_1286 | 0.92325 | -0.11521 | 0.027327 |
| metab_8958 | 1.1238 | 0.16839 | 0.027352 |
| metab_17253 | 1.1174 | 0.16018 | 0.027387 |
| Testosterone Propionate | 1.0548 | 0.07702 | 0.027398 |
| Methylisopelletierine | 1.0355 | 0.050338 | 0.0274 |
| metab_8878 | 0.98391 | -0.023399 | 0.027417 |
| PA(16:1(9Z)/15:0) | 1.1991 | 0.26199 | 0.027443 |
| Cortisol 21-sulfate | 1.4745 | 0.56023 | 0.027502 |
| metab_3134 | 0.98192 | -0.026317 | 0.02757 |
| metab_11754 | 1.0729 | 0.10158 | 0.027595 |
| metab_13096 | 1.0387 | 0.054832 | 0.02764 |
| metab_1310 | 0.94628 | -0.079664 | 0.027767 |
| metab_12107 | 0.74948 | -0.41604 | 0.027896 |
| N-(6-Methoxyquinolin-8-yl)alanine | 1.1291 | 0.17514 | 0.027962 |
| 2-Hexadec-7-enylicosa-8,11-dienedioic acid | 1.0855 | 0.1184 | 0.028081 |
| Erinacine G | 1.1076 | 0.14742 | 0.028129 |
| metab_6896 | 0.99209 | -0.011457 | 0.028136 |
| metab_9128 | 0.69019 | -0.53494 | 0.028242 |
| 4-Hydroxyquinoline | 1.0649 | 0.090751 | 0.02826 |
| metab_4824 | 0.9624 | -0.055287 | 0.02828 |
| metab_8405 | 0.9484 | -0.076428 | 0.028288 |
| metab_11328 | 1.0797 | 0.11065 | 0.028296 |
| metab_1358 | 0.95288 | -0.069637 | 0.028312 |
| metab_17466 | 0.95614 | -0.064709 | 0.02835 |
| metab_8255 | 0.93527 | -0.096548 | 0.028367 |
| metab_14548 | 0.92664 | -0.10992 | 0.028415 |
| metab_10535 | 0.95105 | -0.072409 | 0.028432 |
| O-Acetylcarnitine | 1.0123 | 0.017688 | 0.028496 |
| metab_76 | 1.0985 | 0.13554 | 0.028567 |
| metab_14202 | 0.85858 | -0.21998 | 0.028692 |
| metab_13940 | 1.0761 | 0.10579 | 0.028715 |
| metab_1449 | 0.93881 | -0.091098 | 0.028766 |
| metab_8154 | 1.1473 | 0.19829 | 0.028788 |
| metab_6295 | 0.98621 | -0.020028 | 0.028817 |
| Ile Glu | 1.3131 | 0.39296 | 0.02886 |
| metab_11864 | 0.94367 | -0.083644 | 0.028883 |
| Menthol glucuronide | 1.0954 | 0.13143 | 0.028907 |
| 2-Hydroxy-4-[(1R)-1-hydroxy-8-methyl-6-oxononyl]-3-methyl-2H-furan-5-one | 1.0999 | 0.13737 | 0.028995 |
| Aesculetin | 1.0944 | 0.13009 | 0.028998 |
| metab_8068 | 1.089 | 0.12299 | 0.029082 |
| metab_12996 | 0.96603 | -0.049863 | 0.029097 |
| metab_17669 | 0.98661 | -0.019446 | 0.02911 |
| metab_8384 | 1.0577 | 0.080971 | 0.02911 |
| metab_13258 | 0.96118 | -0.057116 | 0.029113 |
| metab_15406 | 1.0205 | 0.029208 | 0.029127 |
| metab_13090 | 0.93022 | -0.10436 | 0.029159 |
| metab_9382 | 1.1334 | 0.18062 | 0.029242 |
| metab_6389 | 0.88078 | -0.18314 | 0.029293 |
| SM(d18:0/12:0) | 0.97231 | -0.040511 | 0.029309 |
| metab_14225 | 0.91027 | -0.13564 | 0.029313 |
| metab_8426 | 0.8901 | -0.16796 | 0.029354 |
| Daphniphylline | 0.95874 | -0.060786 | 0.029408 |
| metab_11713 | 1.3044 | 0.38337 | 0.029444 |
| metab_3162 | 1.1585 | 0.21221 | 0.029599 |
| metab_11257 | 1.2591 | 0.33244 | 0.029614 |
| metab_10201 | 0.89672 | -0.15726 | 0.029624 |
| metab_12220 | 1.0757 | 0.10531 | 0.029712 |
| metab_3097 | 1.0882 | 0.12197 | 0.029712 |
| metab_12463 | 1.0676 | 0.094335 | 0.029725 |
| metab_10586 | 0.98615 | -0.020125 | 0.029738 |
| PA(8:0/a-17:0) | 1.1235 | 0.16803 | 0.029764 |
| metab_10921 | 1.1373 | 0.18564 | 0.029824 |
| metab_13605 | 0.95082 | -0.072755 | 0.029852 |
| Jasmolone glucoside | 1.0713 | 0.099339 | 0.029864 |
| metab_8013 | 1.149 | 0.2004 | 0.029876 |
| metab_7858 | 0.8869 | -0.17316 | 0.029891 |
| Lippioside I | 1.1181 | 0.1611 | 0.029949 |
| N-acetylaspartate | 1.0196 | 0.027951 | 0.029961 |
| metab_11757 | 1.0609 | 0.085346 | 0.029965 |
| 3-Oxovalproic acid | 1.1075 | 0.14728 | 0.030004 |
| metab_8789 | 1.1888 | 0.24945 | 0.030066 |
| metab_10021 | 0.94445 | -0.082455 | 0.030069 |
| 1-Carboxyethylisoleucine | 1.0316 | 0.044829 | 0.030069 |
| metab_12992 | 0.96837 | -0.04637 | 0.030136 |
| metab_15412 | 1.0292 | 0.041478 | 0.030222 |
| metab_9530 | 0.97434 | -0.0375 | 0.030268 |
| metab_12890 | 0.92079 | -0.11905 | 0.030326 |
| metab_17229 | 1.0749 | 0.10425 | 0.030377 |
| metab_9615 | 0.91409 | -0.12959 | 0.030391 |
| metab_14739 | 0.98823 | -0.017079 | 0.030436 |
| PE(20:3(5Z,8Z,11Z)/18:1(9Z)) | 1.0344 | 0.048801 | 0.03047 |
| metab_13528 | 1.1183 | 0.16125 | 0.030597 |
| metab_13249 | 1.034 | 0.048239 | 0.030686 |
| metab_3153 | 0.95693 | -0.063516 | 0.030733 |
| 3-(Hydroxymethyl)-5,5-diphenylimidazolidine-2,4-dione | 1.0513 | 0.072107 | 0.030741 |
| Penicillamine disulfide | 1.1814 | 0.24053 | 0.030751 |
| metab_13669 | 0.9303 | -0.10423 | 0.030809 |
| metab_3844 | 0.93854 | -0.091517 | 0.030881 |
| metab_3106 | 1.072 | 0.10028 | 0.030951 |
| metab_1265 | 0.9502 | -0.073696 | 0.031075 |
| metab_16403 | 0.94943 | -0.07486 | 0.03108 |
| metab_17354 | 0.97773 | -0.032491 | 0.031106 |
| DG(a-15:0/20:3(5Z,11Z,14Z)-O(8,9)/0:0) | 1.0812 | 0.11264 | 0.03111 |
| Resolvin D2 | 1.1153 | 0.15739 | 0.03114 |
| PE(22:4(7Z,10Z,13Z,16Z)/P-18:0) | 1.0186 | 0.026656 | 0.031162 |
| metab_7164 | 0.96213 | -0.05569 | 0.031195 |
| metab_9075 | 0.9751 | -0.036381 | 0.0313 |
| metab_11435 | 1.0178 | 0.025474 | 0.031303 |
| metab_13838 | 2.2794 | 1.1886 | 0.031475 |
| metab_14031 | 0.97519 | -0.03625 | 0.031497 |
| Fumigaclavine C | 1.0093 | 0.013373 | 0.031523 |
| Deisopropylatrazine | 1.1519 | 0.20397 | 0.031596 |
| metab_14078 | 0.87939 | -0.18542 | 0.031646 |
| metab_3625 | 0.9181 | -0.12328 | 0.03168 |
| metab_2680 | 0.98532 | -0.021335 | 0.031774 |
| [8]-Shogaol | 1.0614 | 0.086022 | 0.031779 |
| metab_8411 | 0.85996 | -0.21766 | 0.031791 |
| metab_11313 | 1.0584 | 0.081819 | 0.031832 |
| metab_15407 | 1.0333 | 0.047213 | 0.031834 |
| 2-(3-Pentylphenyl)acetic acid | 2.2525 | 1.1715 | 0.031918 |
| Coriandrone D | 1.3582 | 0.44165 | 0.031941 |
| metab_5048 | 0.96542 | -0.050767 | 0.032079 |
| LysoPE(P-18:0/0:0) | 0.97949 | -0.029901 | 0.032214 |
| metab_11262 | 0.94441 | -0.08252 | 0.032216 |
| metab_4539 | 1.1179 | 0.16073 | 0.03238 |
| metab_12755 | 0.97574 | -0.035426 | 0.032424 |
| metab_10901 | 0.9599 | -0.059048 | 0.032465 |
| (8S,9S,10S,11S,13S,14S,17R)-11,17-Dihydroxy-17-(2-hydroxyacetyl)-10,13-dimethyl-1,4,5,6,7,8,9,11,12,14,15,16-dodecahydrocyclopenta[a]phenanthrene-2,3-dione | 1.0318 | 0.045119 | 0.032544 |
| metab_15325 | 0.75038 | -0.4143 | 0.032587 |
| metab_495 | 1.1435 | 0.19351 | 0.03269 |
| metab_13656 | 0.86558 | -0.20827 | 0.032714 |
| LysoPC(P-16:0/0:0) | 0.96652 | -0.049136 | 0.032789 |
| metab_11805 | 0.98633 | -0.019853 | 0.032801 |
| Chikusetsusaponin Ia | 0.93339 | -0.099454 | 0.03285 |
| metab_11872 | 1.0589 | 0.082583 | 0.032868 |
| metab_10132 | 0.85907 | -0.21915 | 0.032883 |
| metab_7468 | 1.0205 | 0.029291 | 0.033005 |
| metab_183 | 0.95325 | -0.069071 | 0.033008 |
| (S)-10,16-Dihydroxyhexadecanoic acid | 1.036 | 0.050968 | 0.03303 |
| metab_7912 | 1.0602 | 0.084344 | 0.033057 |
| (6'-Hydroxy-3-oxospiro[2-benzofuran-1,9'-xanthene]-3'-yl) acetate | 1.1343 | 0.18177 | 0.033177 |
| metab_13964 | 0.89953 | -0.15275 | 0.033201 |
| metab_13701 | 0.94029 | -0.088827 | 0.03323 |
| metab_12318 | 0.93894 | -0.090888 | 0.033295 |
| metab_654 | 0.99294 | -0.010222 | 0.033314 |
| metab_4382 | 1.0153 | 0.021884 | 0.033363 |
| metab_17692 | 0.9776 | -0.032689 | 0.033373 |
| metab_8884 | 0.9214 | -0.1181 | 0.033395 |
| metab_3521 | 1.186 | 0.24615 | 0.033688 |
| metab_17663 | 0.95178 | -0.071299 | 0.033711 |
| metab_1504 | 0.96735 | -0.047887 | 0.033724 |
| metab_13936 | 0.8293 | -0.27004 | 0.03374 |
| metab_6571 | 1.0151 | 0.02164 | 0.033811 |
| metab_12735 | 0.97726 | -0.033188 | 0.033839 |
| metab_1136 | 0.9625 | -0.055134 | 0.033887 |
| metab_4998 | 1.1145 | 0.15634 | 0.033888 |
| Phenylalanylproline | 1.0349 | 0.049538 | 0.033929 |
| N-formylanthranilic acid | 1.0449 | 0.06332 | 0.033941 |
| metab_2678 | 1.0487 | 0.068547 | 0.033945 |
| Calcitroic acid | 1.0447 | 0.063035 | 0.03397 |
| N-Acetyl-DL-tryptophan | 1.1258 | 0.17091 | 0.034022 |
| metab_10860 | 0.96567 | -0.050394 | 0.034159 |
| metab_5513 | 0.98396 | -0.023331 | 0.034214 |
| Thiodiacetic Acid | 1.21 | 0.27498 | 0.034249 |
| metab_24 | 1.0746 | 0.10385 | 0.03426 |
| metab_13620 | 1.2414 | 0.312 | 0.034288 |
| metab_14804 | 0.9642 | -0.052591 | 0.034427 |
| metab_2725 | 1.0218 | 0.03118 | 0.034437 |
| Deoxyribose | 1.0297 | 0.042283 | 0.034495 |
| metab_7165 | 1.0099 | 0.01424 | 0.034523 |
| metab_9519 | 1.1055 | 0.14468 | 0.034566 |
| metab_16850 | 1.1203 | 0.16385 | 0.034612 |
| Dihydrocaffeic acid 3-O-glucuronide | 1.1306 | 0.17705 | 0.034616 |
| metab_17165 | 1.0761 | 0.10587 | 0.034619 |
| metab_16616 | 1.1237 | 0.16823 | 0.034647 |
| metab_9396 | 0.67274 | -0.57187 | 0.03465 |
| metab_3048 | 1.0394 | 0.055805 | 0.034669 |
| metab_2597 | 0.98678 | -0.019198 | 0.034705 |
| metab_7760 | 1.0331 | 0.046971 | 0.034789 |
| metab_12148 | 0.93374 | -0.098906 | 0.034859 |
| metab_8998 | 0.93395 | -0.098575 | 0.034901 |
| metab_15337 | 1.0393 | 0.055597 | 0.034921 |
| metab_7478 | 1.0216 | 0.030799 | 0.035049 |
| Estradiol cypionate | 1.0792 | 0.10992 | 0.035075 |
| metab_1119 | 1.0283 | 0.040255 | 0.035096 |
| metab_8107 | 0.79529 | -0.33045 | 0.03512 |
| metab_11367 | 1.1458 | 0.19641 | 0.03523 |
| metab_12216 | 1.0431 | 0.06082 | 0.035273 |
| metab_14479 | 0.92018 | -0.12002 | 0.03531 |
| metab_3513 | 0.98584 | -0.020568 | 0.035315 |
| metab_10062 | 1.1369 | 0.18509 | 0.035371 |
| metab_12196 | 1.199 | 0.26182 | 0.035393 |
| Oleoyl-L-Carnitine | 1.0234 | 0.033376 | 0.035474 |
| N-Linoleoyl Tyrosine | 1.031 | 0.044107 | 0.035478 |
| metab_8430 | 0.99023 | -0.014165 | 0.035488 |
| metab_849 | 0.95335 | -0.068917 | 0.035528 |
| metab_14177 | 1.3074 | 0.38675 | 0.035534 |
| 5-(Hydroxymethyl)-4-methoxy-2(5H)-furanone | 1.014 | 0.020023 | 0.035574 |
| metab_14303 | 1.1556 | 0.20864 | 0.035645 |
| metab_14613 | 0.92991 | -0.10483 | 0.035733 |
| metab_14280 | 1.1591 | 0.21302 | 0.035896 |
| Tyramine glucuronide | 0.88628 | -0.17416 | 0.036007 |
| metab_4793 | 0.98438 | -0.022717 | 0.036085 |
| metab_649 | 1.0267 | 0.038029 | 0.036176 |
| metab_2660 | 0.99034 | -0.01401 | 0.036208 |
| metab_2971 | 1.1443 | 0.19447 | 0.036322 |
| Gibberellin A15 | 1.1087 | 0.14885 | 0.03644 |
| metab_3793 | 1.0652 | 0.091188 | 0.036473 |
| metab_9969 | 0.95095 | -0.072564 | 0.036482 |
| metab_11645 | 1.0422 | 0.059576 | 0.036564 |
| Fenbuconazole | 1.094 | 0.12968 | 0.036738 |
| metab_13739 | 1.0207 | 0.029489 | 0.036753 |
| metab_17312 | 0.9108 | -0.13479 | 0.036765 |
| Rotenone | 1.1281 | 0.17386 | 0.036769 |
| Dopamine 4-sulfate | 1.0636 | 0.089012 | 0.036788 |
| metab_12864 | 0.98788 | -0.017596 | 0.03682 |
| metab_12884 | 0.92617 | -0.11065 | 0.036829 |
| metab_566 | 1.0422 | 0.059678 | 0.036839 |
| metab_8185 | 0.91013 | -0.13586 | 0.036969 |
| metab_17629 | 1.0555 | 0.077904 | 0.037026 |
| 1-Methylnicotinamide | 1.0453 | 0.06393 | 0.037043 |
| metab_14166 | 0.97593 | -0.035146 | 0.037069 |
| Derhamnosylmaysin | 0.95456 | -0.067092 | 0.037069 |
| metab_3039 | 1.0785 | 0.10907 | 0.037202 |
| metab_11485 | 1.0727 | 0.10129 | 0.037231 |
| metab_14822 | 0.92013 | -0.12009 | 0.037235 |
| metab_12132 | 1.0302 | 0.042904 | 0.037235 |
| metab_9078 | 1.1318 | 0.17867 | 0.037268 |
| metab_2331 | 1.0445 | 0.062833 | 0.03737 |
| metab_10091 | 0.9241 | -0.11388 | 0.037457 |
| metab_8130 | 1.1047 | 0.14368 | 0.037489 |
| Polyoxyethylene 40 monostearate | 1.0112 | 0.016072 | 0.0375 |
| 3-[Bis(4-methoxyphenyl)methylidene]-1H-indol-2-one | 1.2332 | 0.30243 | 0.037583 |
| metab_12057 | 0.95923 | -0.060044 | 0.037661 |
| metab_10502 | 0.98878 | -0.01628 | 0.037685 |
| metab_10581 | 0.9665 | -0.049163 | 0.037731 |
| metab_5084 | 1.201 | 0.26429 | 0.037795 |
| Mono-(2-ethyl-5-carboxypentyl) phthalate | 1.0537 | 0.075436 | 0.037893 |
| metab_7854 | 0.96616 | -0.049665 | 0.037971 |
| metab_8888 | 1.1399 | 0.18894 | 0.037983 |
| metab_14183 | 1.1208 | 0.16447 | 0.038 |
| metab_15448 | 0.93508 | -0.096831 | 0.038004 |
| N-(3,5-Dihydroxy-4-azatetracyclo[5.3.2.02,6.08,10]dodeca-2,5,11-trien-4-yl)-4-(trifluoromethyl)benzamide | 1.2216 | 0.28873 | 0.038019 |
| Perindoprilat glucuronide | 1.0539 | 0.075784 | 0.03805 |
| metab_9527 | 1.0344 | 0.048742 | 0.038088 |
| Islatravir | 0.92807 | -0.10769 | 0.038105 |
| Hydroxyfluoroprednisolone butyrate | 1.0306 | 0.043481 | 0.038122 |
| metab_11355 | 0.97609 | -0.034913 | 0.038184 |
| metab_13682 | 1.0169 | 0.024125 | 0.038204 |
| 4-(2-Aminophenyl)-2,4-dioxobutanoic acid | 1.0775 | 0.10769 | 0.038212 |
| metab_2094 | 1.1172 | 0.15988 | 0.038224 |
| metab_4249 | 1.0811 | 0.11246 | 0.038408 |
| metab_4466 | 1.0904 | 0.12489 | 0.038429 |
| metab_5558 | 1.0653 | 0.091326 | 0.038461 |
| metab_4564 | 0.67816 | -0.56029 | 0.038471 |
| metab_17274 | 1.2055 | 0.26968 | 0.038529 |
| metab_837 | 0.84173 | -0.24858 | 0.038531 |
| PA(8:0/10:0) | 0.92136 | -0.11816 | 0.038676 |
| Agavoside A | 1.0986 | 0.13573 | 0.038677 |
| metab_3711 | 0.93925 | -0.090417 | 0.038704 |
| metab_1379 | 0.97991 | -0.029283 | 0.038728 |
| metab_8278 | 0.99248 | -0.010885 | 0.038794 |
| metab_1472 | 0.96436 | -0.052351 | 0.038829 |
| metab_9201 | 0.98878 | -0.016279 | 0.038834 |
| metab_10665 | 0.94841 | -0.07642 | 0.038901 |
| PS(20:0/15:0) | 0.96798 | -0.046945 | 0.038952 |
| 2,3-Dimethyl-2-cyclohexen-1-one | 1.0452 | 0.063738 | 0.038974 |
| metab_11469 | 1.5141 | 0.59847 | 0.039015 |
| metab_1197 | 1.0809 | 0.11219 | 0.039022 |
| metab_2867 | 1.0922 | 0.12718 | 0.039219 |
| metab_9314 | 1.2436 | 0.31455 | 0.039266 |
| metab_17451 | 0.89803 | -0.15516 | 0.039322 |
| metab_1424 | 1.5261 | 0.6099 | 0.039404 |
| metab_8587 | 1.0638 | 0.089227 | 0.039474 |
| metab_2039 | 1.0407 | 0.057518 | 0.039549 |
| metab_13746 | 1.0453 | 0.06398 | 0.039642 |
| metab_3827 | 1.061 | 0.085363 | 0.039651 |
| metab_310 | 1.0678 | 0.094651 | 0.039664 |
| metab_13840 | 1.1923 | 0.25378 | 0.039686 |
| Pantoprazole sulfide | 1.0841 | 0.11656 | 0.039687 |
| metab_3490 | 0.68458 | -0.5467 | 0.03982 |
| 1-Acetylindole | 1.0245 | 0.034954 | 0.03983 |
| metab_3925 | 1.0786 | 0.10921 | 0.039847 |
| metab_8296 | 0.93503 | -0.096917 | 0.039867 |
| metab_514 | 1.0386 | 0.054631 | 0.039881 |
| metab_3225 | 1.0142 | 0.020366 | 0.039883 |
| metab_12258 | 1.1058 | 0.14515 | 0.039908 |
| metab_4265 | 1.0239 | 0.034062 | 0.039945 |
| metab_16167 | 0.92076 | -0.11911 | 0.040029 |
| metab_15161 | 1.7581 | 0.81399 | 0.040122 |
| metab_13256 | 0.96395 | -0.052972 | 0.040123 |
| metab_9557 | 0.89089 | -0.16668 | 0.040156 |
| metab_7079 | 1.0389 | 0.055067 | 0.040313 |
| metab_6771 | 1.0295 | 0.042007 | 0.040315 |
| metab_4258 | 1.1643 | 0.21945 | 0.04032 |
| (3a,5b,7a)-23-Carboxy-7-hydroxy-24-norcholan-3-yl-b-D-Glucopyranosiduronic acid | 1.127 | 0.17246 | 0.040338 |
| metab_14027 | 0.91353 | -0.13047 | 0.040362 |
| metab_8493 | 1.0717 | 0.099901 | 0.040411 |
| metab_9852 | 1.1489 | 0.2002 | 0.04045 |
| metab_8986 | 1.1452 | 0.1956 | 0.040452 |
| metab_5646 | 0.73662 | -0.44102 | 0.040455 |
| metab_13451 | 1.0807 | 0.11191 | 0.040474 |
| metab_7252 | 1.0721 | 0.10038 | 0.040482 |
| metab_17111 | 0.93516 | -0.09672 | 0.040483 |
| Arginylmethionine | 1.3206 | 0.40121 | 0.040539 |
| metab_14727 | 0.88885 | -0.16998 | 0.040598 |
| metab_16184 | 1.0591 | 0.082779 | 0.040639 |
| 6-Hydroxy-1H-indole-3-acetamide | 1.1845 | 0.24426 | 0.040652 |
| metab_10139 | 1.1407 | 0.1899 | 0.040689 |
| metab_13116 | 1.2495 | 0.32139 | 0.040699 |
| metab_13612 | 0.95484 | -0.066675 | 0.040721 |
| metab_14318 | 1.265 | 0.33912 | 0.040739 |
| (3-Methoxy-4-hydroxyphenyl)ethylene glycol sulfate | 1.0357 | 0.050563 | 0.040746 |
| metab_10958 | 0.98654 | -0.019554 | 0.040764 |
| metab_13965 | 0.91392 | -0.12986 | 0.040817 |
| metab_10797 | 1.0549 | 0.077151 | 0.040869 |
| metab_8535 | 0.93847 | -0.091616 | 0.040877 |
| metab_16997 | 0.98401 | -0.023252 | 0.040966 |
| metab_12162 | 1.1118 | 0.15284 | 0.040969 |
| metab_9423 | 1.3749 | 0.45932 | 0.040977 |
| metab_16629 | 0.97082 | -0.042726 | 0.040984 |
| metab_13945 | 0.90913 | -0.13744 | 0.040998 |
| metab_11726 | 1.3318 | 0.41336 | 0.041 |
| metab_2666 | 1.0905 | 0.12494 | 0.041307 |
| metab_15339 | 1.1092 | 0.14951 | 0.041322 |
| GPEtn(20:4/18:1) | 1.0251 | 0.035707 | 0.041339 |
| metab_16389 | 1.0496 | 0.069848 | 0.041389 |
| metab_10825 | 0.89518 | -0.15975 | 0.041413 |
| metab_10093 | 0.89531 | -0.15954 | 0.041475 |
| metab_8227 | 0.92919 | -0.10596 | 0.041483 |
| metab_10869 | 1.0525 | 0.073778 | 0.041592 |
| metab_16041 | 1.2158 | 0.28188 | 0.041625 |
| metab_8001 | 1.0273 | 0.038921 | 0.041823 |
| metab_3541 | 1.0564 | 0.079108 | 0.041842 |
| metab_4163 | 0.98263 | -0.025286 | 0.041924 |
| metab_17036 | 1.0488 | 0.068738 | 0.042002 |
| metab_574 | 1.0072 | 0.010375 | 0.04218 |
| metab_13641 | 0.94972 | -0.074422 | 0.042196 |
| metab_16574 | 1.0547 | 0.076795 | 0.042209 |
| metab_12444 | 1.0515 | 0.072473 | 0.042234 |
| metab_12892 | 1.055 | 0.077308 | 0.042261 |
| Coformycin | 1.028 | 0.03981 | 0.042334 |
| metab_8026 | 0.9059 | -0.14257 | 0.042372 |
| Albanin G | 1.4901 | 0.57546 | 0.04242 |
| 1,6-anhydro-N-acetyl-beta-muramate | 1.0332 | 0.047175 | 0.042501 |
| metab_10315 | 1.1231 | 0.16752 | 0.04251 |
| metab_3128 | 1.0318 | 0.045197 | 0.042529 |
| metab_12494 | 0.97737 | -0.033024 | 0.04254 |
| DG(18:2(9Z,12Z)/15:0/0:0) | 1.0577 | 0.080921 | 0.04258 |
| metab_10371 | 0.97649 | -0.034321 | 0.042665 |
| P-Hydroxyubenimex | 1.0674 | 0.094085 | 0.042673 |
| Icariside D1 | 1.0364 | 0.051527 | 0.04279 |
| metab_17016 | 0.99122 | -0.012721 | 0.042831 |
| N-Arachidonoylglycine | 1.0216 | 0.030785 | 0.042967 |
| metab_12224 | 1.1666 | 0.22236 | 0.043024 |
| metab_15614 | 0.90883 | -0.13792 | 0.043051 |
| metab_13779 | 1.0783 | 0.10882 | 0.043062 |
| metab_15481 | 0.97543 | -0.035885 | 0.043081 |
| metab_7539 | 1.04 | 0.056628 | 0.043096 |
| metab_2545 | 0.98916 | -0.015729 | 0.043162 |
| DG(PGD2/a-17:0/0:0) | 1.0324 | 0.045958 | 0.043165 |
| metab_10436 | 1.1219 | 0.16592 | 0.04326 |
| metab_3772 | 1.021 | 0.029973 | 0.043362 |
| metab_12900 | 0.97511 | -0.036364 | 0.043364 |
| metab_195 | 1.1373 | 0.18555 | 0.043375 |
| metab_8773 | 0.92792 | -0.10793 | 0.043447 |
| metab_14664 | 0.88237 | -0.18055 | 0.043576 |
| metab_15238 | 0.9138 | -0.13004 | 0.043657 |
| metab_9608 | 2.9562 | 1.5637 | 0.043682 |
| metab_15338 | 0.90757 | -0.13992 | 0.043738 |
| metab_8078 | 0.95456 | -0.067089 | 0.043742 |
| metab_15482 | 0.98242 | -0.025581 | 0.043758 |
| metab_13139 | 1.1173 | 0.15996 | 0.043787 |
| Threonolactone | 0.91922 | -0.12151 | 0.043839 |
| metab_4370 | 1.0305 | 0.043363 | 0.043846 |
| metab_14688 | 1.1089 | 0.14917 | 0.044005 |
| DNOC | 0.97029 | -0.043519 | 0.044032 |
| metab_8302 | 0.96414 | -0.052689 | 0.04412 |
| Hydroxymethyl cimetidine | 0.8651 | -0.20906 | 0.044214 |
| PA(i-12:0/PGF1alpha) | 0.97327 | -0.039081 | 0.044341 |
| metab_15684 | 0.97114 | -0.042254 | 0.044405 |
| metab_10175 | 0.90443 | -0.14491 | 0.044419 |
| 6-Gingerol | 1.5188 | 0.6029 | 0.044475 |
| metab_8135 | 0.89874 | -0.15403 | 0.044512 |
| metab_1477 | 1.067 | 0.093512 | 0.044535 |
| metab_4637 | 1.0268 | 0.038179 | 0.044694 |
| metab_717 | 0.7899 | -0.34026 | 0.044706 |
| metab_8917 | 0.97275 | -0.039865 | 0.044748 |
| metab_9026 | 2.1516 | 1.1054 | 0.044794 |
| metab_9677 | 0.97578 | -0.035378 | 0.044838 |
| metab_15304 | 1.1298 | 0.17607 | 0.04484 |
| metab_13832 | 1.1004 | 0.13798 | 0.044848 |
| metab_14997 | 0.89325 | -0.16287 | 0.044873 |
| SAICAR | 0.90677 | -0.14119 | 0.044873 |
| metab_3283 | 0.97621 | -0.03473 | 0.044936 |
| metab_7676 | 0.95978 | -0.059223 | 0.045196 |
| Sagopilone | 0.84308 | -0.24627 | 0.045287 |
| metab_1285 | 1.0614 | 0.086034 | 0.045293 |
| metab_13084 | 0.91363 | -0.13032 | 0.045307 |
| PC(16:0/22:5(7Z,10Z,13Z,16Z,19Z)) | 1.0073 | 0.010533 | 0.04531 |
| metab_13397 | 1.0196 | 0.027951 | 0.045382 |
| metab_5395 | 0.988 | -0.017419 | 0.045455 |
| metab_8231 | 1.1725 | 0.22959 | 0.045473 |
| metab_3645 | 0.98611 | -0.020177 | 0.045483 |
| Glycinoeclepin B | 0.93041 | -0.10406 | 0.045513 |
| metab_1791 | 0.99114 | -0.012833 | 0.045517 |
| metab_2747 | 0.96219 | -0.0556 | 0.045573 |
| metab_8057 | 1.038 | 0.05379 | 0.04561 |
| (3Z)-2-Propylpent-3-enoic acid | 1.0262 | 0.037275 | 0.045708 |
| metab_12998 | 0.97417 | -0.037758 | 0.045735 |
| metab_14037 | 0.88299 | -0.17953 | 0.045763 |
| metab_8325 | 0.93289 | -0.10022 | 0.045841 |
| metab_7323 | 1.1943 | 0.25613 | 0.045864 |
| metab_9618 | 0.97052 | -0.043176 | 0.045933 |
| metab_5642 | 0.97509 | -0.036397 | 0.046007 |
| metab_10374 | 0.97377 | -0.038349 | 0.046009 |
| metab_5226 | 1.0807 | 0.112 | 0.046115 |
| CDP-DG(i-22:0/18:3(10,12,15)-OH(9)) | 0.79902 | -0.3237 | 0.046116 |
| Pimelic Acid | 1.0223 | 0.031884 | 0.046122 |
| metab_7147 | 0.89905 | -0.15352 | 0.046223 |
| metab_7236 | 1.1411 | 0.19039 | 0.046278 |
| metab_8784 | 0.86961 | -0.20156 | 0.04634 |
| metab_5292 | 0.96549 | -0.050674 | 0.046347 |
| Folinic acid | 1.0432 | 0.060971 | 0.046376 |
| metab_13548 | 0.94814 | -0.076832 | 0.0464 |
| metab_15500 | 1.1249 | 0.16983 | 0.046411 |
| metab_1867 | 0.98193 | -0.026312 | 0.046424 |
| metab_10377 | 0.97722 | -0.033241 | 0.046438 |
| metab_12997 | 1.0102 | 0.014612 | 0.046482 |
| Naloxazone | 1.0984 | 0.13534 | 0.046497 |
| metab_13952 | 1.0081 | 0.011615 | 0.046501 |
| Eddha | 1.0557 | 0.078137 | 0.046506 |
| metab_15194 | 0.958 | -0.061905 | 0.046518 |
| Aminohippuric acid | 1.0756 | 0.10508 | 0.046533 |
| metab_6053 | 0.92778 | -0.10814 | 0.046702 |
| metab_3507 | 0.98998 | -0.01453 | 0.046787 |
| metab_11246 | 1.0999 | 0.13732 | 0.046858 |
| (-)-alpha-Bisabolol | 1.0277 | 0.039435 | 0.046859 |
| metab_2370 | 0.79554 | -0.32999 | 0.046867 |
| metab_9673 | 0.99192 | -0.0117 | 0.046873 |
| metab_11335 | 1.0448 | 0.063161 | 0.046878 |
| metab_2234 | 1.1482 | 0.19939 | 0.04699 |
| metab_5397 | 1.0518 | 0.072813 | 0.047053 |
| 3-Methoxyanthranilate | 1.1166 | 0.15915 | 0.04708 |
| metab_6953 | 1.0134 | 0.01918 | 0.047208 |
| metab_9655 | 0.95538 | -0.065854 | 0.04739 |
| metab_10087 | 0.87299 | -0.19596 | 0.047404 |
| 2-Pyrimidine Acetic Acid | 1.1163 | 0.15874 | 0.047429 |
| metab_10693 | 0.98767 | -0.017896 | 0.047521 |
| Guanylin | 0.69994 | -0.51469 | 0.047525 |
| metab_8463 | 0.97029 | -0.043511 | 0.047533 |
| 2-Lysophosphatidylcholine | 0.97106 | -0.042362 | 0.047557 |
| metab_1324 | 0.90298 | -0.14723 | 0.047595 |
| metab_11162 | 1.0303 | 0.043124 | 0.047599 |
| metab_10855 | 0.9688 | -0.04573 | 0.047714 |
| metab_11712 | 1.0881 | 0.12182 | 0.047989 |
| metab_1727 | 0.97205 | -0.040903 | 0.048045 |
| metab_5097 | 1.1133 | 0.15478 | 0.048119 |
| metab_10644 | 0.96378 | -0.053218 | 0.048121 |
| metab_11360 | 1.4181 | 0.50401 | 0.048223 |
| metab_9478 | 0.92699 | -0.10938 | 0.048271 |
| metab_4002 | 1.1689 | 0.22512 | 0.048332 |
| metab_9266 | 1.0665 | 0.092833 | 0.048528 |
| metab_14629 | 1.0146 | 0.020898 | 0.048585 |
| metab_15186 | 0.94962 | -0.07458 | 0.048614 |
| metab_13757 | 1.1938 | 0.25558 | 0.048647 |
| metab_2041 | 0.92337 | -0.11502 | 0.048665 |
| metab_3716 | 0.91872 | -0.1223 | 0.048674 |
| metab_10034 | 0.96922 | -0.045108 | 0.048724 |
| FAHFA(16:1(9Z)/13-O-16:0) | 1.0609 | 0.085317 | 0.048745 |
| 1-Methylguanosine | 1.0399 | 0.056407 | 0.048757 |
| metab_14720 | 0.95893 | -0.060496 | 0.04896 |
| metab_12761 | 0.99042 | -0.013887 | 0.049006 |
| metab_6515 | 0.89869 | -0.15411 | 0.049015 |
| metab_394 | 1.0575 | 0.080637 | 0.049045 |
| metab_8745 | 1.3695 | 0.4536 | 0.049183 |
| metab_1350 | 0.97855 | -0.031284 | 0.049191 |
| metab_14594 | 0.92099 | -0.11874 | 0.049253 |
| metab_10392 | 0.9687 | -0.045879 | 0.049277 |
| metab_364 | 1.0445 | 0.062851 | 0.049352 |
| metab_9997 | 1.0408 | 0.05763 | 0.049459 |
| metab_13666 | 0.94927 | -0.075107 | 0.049466 |
| metab_9674 | 1.2911 | 0.36866 | 0.049626 |
| metab_17262 | 0.85196 | -0.23113 | 0.049634 |
| metab_886 | 0.98092 | -0.027788 | 0.049694 |
| metab_2273 | 1.041 | 0.058019 | 0.049747 |
| SM(d18:1/14:1(9Z)) | 0.97646 | -0.034365 | 0.049761 |
| metab_773 | 1.0579 | 0.08122 | 0.049788 |
| metab_13093 | 1.0324 | 0.045954 | 0.0498 |
| metab_10498 | 0.98453 | -0.022486 | 0.049887 |
| Hymenoxon | 1.0474 | 0.06687 | 0.049891 |
| metab_554 | 1.0251 | 0.035764 | 0.049975 |

| Table S6.The differential metabolites between ND and NND groups (adjusted). | | |
| --- | --- | --- |
|  | P value | FDR |
| Linolenic Acid | 2.9877e-11 | 2.2491e-07 |
| metab_5468 | 2.9877e-11 | 2.2491e-07 |
| Octadeca-9,12-dienal | 5.8851e-11 | 2.9536e-07 |
| Sclareol oxide | 2.1037e-10 | 7.9181e-07 |
| metab_5443 | 5.1393e-10 | 1.4732e-06 |
| 3-Gonal | 5.9367e-10 | 1.4732e-06 |
| Gamma-Sanshool | 6.8493e-10 | 1.4732e-06 |
| (Z)-9-Cycloheptadecen-1-one | 1.0442e-09 | 1.9652e-06 |
| Dodemorph | 2.061e-09 | 3.4477e-06 |
| metab_2601 | 2.6847e-09 | 4.0421e-06 |
| Arctiol | 3.483e-09 | 4.7673e-06 |
| metab_6630 | 1.072e-08 | 1.345e-05 |
| metab_2258 | 2.7448e-08 | 3.1789e-05 |
| (R)-3,4-Dihydro-2-methyl-2-(4,8,12-trimethyl-3,7,11-tridecatrienyl)-2H-1-benzopyran-6-ol | 1.2665e-07 | 0.0001362 |
| metab_6693 | 4.2385e-07 | 0.00042543 |
| metab_6541 | 4.6692e-07 | 0.00043937 |
| metab_4815 | 9.051e-07 | 0.00075707 |
| metab_8008 | 9.051e-07 | 0.00075707 |
| Betamethasone phosphate | 6.6831e-06 | 0.0052958 |
| 1,4-Naphthoquinone | 7.2494e-06 | 0.0054573 |
| metab_11697 | 8.5188e-06 | 0.0061076 |
| Food orange 7 | 9.2286e-06 | 0.0063157 |
| metab_4834 | 1.4786e-05 | 0.0096789 |
| metab_47 | 1.7244e-05 | 0.010818 |
| Glyceraldehyde | 2.0079e-05 | 0.012093 |
| metab_12193 | 2.5154e-05 | 0.014566 |
| metab_11357 | 3.3785e-05 | 0.018839 |
| metab_12188 | 4.5102e-05 | 0.024252 |
| Tuberose lactone | 5.5797e-05 | 0.028968 |
| metab_6819 | 7.3726e-05 | 0.037 |
| metab_2684 | 9.0522e-05 | 0.043964 |
|  |  |  |

| Table S7.The correlation between linolenic acid and the five domains of the ASQ-3 score. | | |
| --- | --- | --- |
|  | Adjusted R^2^ | P value |
| ASQ total score |  |  |
| Unadjusted model | 0.281 | <0.001 |
| Adjusted model | 0.313 | 0.009 |
| Communication |  |  |
| Unadjusted model | 0.014 | 0.34 |
| Adjusted model | 0.052 | 0.98 |
| Gross motor |  |  |
| Unadjusted model | 0.245 | <0.001 |
| Adjusted model | 0.262 | 0.014 |
| Fine motor |  |  |
| Unadjusted model | 0.126 | 0.002 |
| Adjusted model | 0.146 | 0.160 |
| Problem solving |  |  |
| Unadjusted model | 0.149 | <0.001 |
| Adjusted model | 0.177 | 0.097 |
| Personal social |  |  |
| Unadjusted model | 0.253 | <0.001 |
| Adjusted model | 0.251 | 0.003 |
| Adjust for age, BMI z score, sex, and SpO2. | | |
